# Clinical Validation of a Cell-Free DNA Fragmentome Assay for Augmentation of Lung Cancer Early Detection

**DOI:** 10.1158/2159-8290.CD-24-0519

**Published:** 2024-06-06

**Authors:** Peter J. Mazzone, Peter B. Bach, Jacob Carey, Caitlin A. Schonewolf, Katalin Bognar, Manmeet S. Ahluwalia, Marcia Cruz-Correa, David Gierada, Sonali Kotagiri, Kathryn Lloyd, Fabien Maldonado, Jesse D. Ortendahl, Lecia V. Sequist, Gerard A. Silvestri, Nichole Tanner, Jeffrey C. Thompson, Anil Vachani, Kwok-Kin Wong, Ali H. Zaidi, Joseph Catallini, Ariel Gershman, Keith Lumbard, Laurel K. Millberg, Jeff Nawrocki, Carter Portwood, Aakanksha Rangnekar, Carolina Campos Sheridan, Niti Trivedi, Tony Wu, Yuhua Zong, Lindsey Cotton, Allison Ryan, Christopher Cisar, Alessandro Leal, Nicholas Dracopoli, Robert B. Scharpf, Victor E. Velculescu, Luke R. G. Pike

**Affiliations:** 1 Cleveland Clinic, Cleveland, Ohio.; 2 DELFI Diagnostics, Baltimore, Maryland.; 3 Medicus Economics, LLC, Formerly PHAR, San Francisco, California.; 4 Miami Cancer Institute, Baptist Health South Florida, Miami, Florida.; 5 Pan American Center for Oncology, San Juan, Puerto Rico.; 6 Washington University at St. Louis, St. Louis, Missouri.; 7 Centura Health, Aurora, Colorado.; 8 Vanderbilt Health, Nashville, Tennessee.; 9 Stratevi, Formerly PHAR, San Francisco, California.; 10 Massachusetts General Hospital, Boston, Massachusetts.; 11 Medical University of South Carolina, Charleston, South Carolina.; 12 Department of Veterans Affairs, Charleston, South Carolina.; 13 Division of Pulmonary, Allergy and Critical Care Medicine, Thoracic Oncology Group, Department of Medicine, Perelman School of Medicine, University of Pennsylvania, Philadelphia, Pennsylvania.; 14 New York University Langone Health, New York, New York.; 15 Allegheny Health Network, Pittsburgh, Pennsylvania.; 16 Sidney Kimmel Comprehensive Cancer Center, Johns Hopkins University School of Medicine, Baltimore, Maryland.; 17 Memorial Sloan Kettering Cancer Center, New York, New York.

## Abstract

Lung cancer screening via annual low-dose computed tomography has poor adoption. We conducted a prospective case–control study among 958 individuals eligible for lung cancer screening to develop a blood-based lung cancer detection test that when positive is followed by a low-dose computed tomography. Changes in genome-wide cell-free DNA fragmentation profiles (fragmentomes) in peripheral blood reflected genomic and chromatin characteristics of lung cancer. We applied machine learning to fragmentome features to identify individuals who were more or less likely to have lung cancer. We trained the classifier using 576 cases and controls from study samples and validated it in a held-out group of 382 cases and controls. The validation demonstrated high sensitivity for lung cancer and consistency across demographic groups and comorbid conditions. Applying test performance to the screening eligible population in a 5-year model with modest utilization assumptions suggested the potential to prevent thousands of lung cancer deaths.

**Significance:**

Lung cancer screening has poor adoption. Our study describes the development and validation of a novel blood-based lung cancer screening test utilizing a highly affordable, low-coverage genome-wide sequencing platform to analyze cell-free DNA fragmentation patterns. The test could improve lung cancer screening rates leading to substantial public health benefits.

*See related commentary by Haber and Skates, p. 2025*

## Teaser

A high-sensitivity, low-cost cell-free DNA fragmentome test is validated in the population eligible for lung cancer screening, with the potential to prevent thousands of deaths if used to close screening shortfalls.

## Introduction

Lung cancer is the number one cancer killer of men and women in the United States, accounting for more than 125,000 deaths per year in the US and nearly 1.8 million deaths per year globally (1–3). Annual screening for lung cancer among high-risk individuals is highly effective at detecting early-stage cancer. Two randomized controlled trials of chest low-dose computed tomography (LDCT) conducted in well-defined, high-risk populations, demonstrated lung cancer mortality reductions of 20% to 24% (4–6). These data led to wide endorsement of lung cancer screening in the United States, initially encompassing individuals aged 55 to 80 who had accumulated 30 pack-years or more of smoking and had quit for no more than 15 years (6, 7). In 2021, the United States Preventive Services Task Force (USPSTF) expanded eligibility for lung cancer screening to individuals beginning at age 50 and with 20 pack-years of smoking history who had quit within 15 years, and in 2023, the American Cancer Society further expanded the guidelines to encompass individuals with similar risk factors irrespective of the duration of smoking cessation (8, 9).

Adoption of LDCT for lung cancer screening in the United States, despite guideline recommendations and insurance coverage, has been poor (1, 8, 10, 11). Estimated rates of lung screening annually range from 5% to 10%—lower than for other recommended cancer screenings (1, 12, 13). Of those who have undergone LDCT screening, annual return rates are also low compared with screening for other cancers (14). Among the first million people screened in the United States, only 22.3% returned for the next annual LDCT screening (15).

Patient barriers to screening include insufficient awareness of screening benefits, concerns for radiation-related cancer due to repetitive CT imaging, heightened anxiety when lung abnormalities are detected, and limited access (16, 17). Other obstacles include insufficient documentation of smoking history needed to determine eligibility, a lack of standardized protocols for screening implementation, and limited availability of specialist follow-up and same-day point-of-care scheduling.

Some share of these obstacles might be ameliorated if there were a blood–based screening test for lung cancer that could be employed among eligible individuals who were not being screened routinely with LDCT. Such a clinical application could increase overall screening rates in much the same manner as the expansion of colorectal cancer screening tools improved overall early detection. The Fecal Immunochemical Test (FIT) test as an initial evaluation for colorectal cancer is one example of a tool that has expanded colorectal cancer screening. When the test is positive, the next “reflex” test is a screening colonoscopy. The proposed lung cancer early detection blood test would play a similar role—an initial evaluation tool that when positive would be followed by a screening LDCT (13, 18, 19). The clinical requirements of the lung cancer test would also parallel the features of the FIT test: low cost, logistically simple so as to enable reaching populations that struggle to access LDCT screening, and high sensitivity (the ability for the test to detect the disease when it is present) including for early-stage disease. The test’s specificity (the frequency with which the test returns a “negative” result when the disease is not present) is not as important as its sensitivity in this clinical context. As an adjunct screening test, false positive results (the frequency of which is the opposite of the test’s specificity) lead to the established standard of care LDCT screening.

Several proof-of-concept studies have explored blood-based detection of lung cancer. Early efforts relying on targeted interrogation of the genome have had poor sensitivity for early-stage disease (20–22). Low pass–whole genome sequencing to evaluate genome-wide cell-free DNA (cfDNA) fragmentation profiles, in contrast, has shown promise (23, 24). During the analyses of case–control and prospective cohorts used to train and evaluate machine learning–based classifiers of cfDNA fragmentation, high sensitivity for early-stage cancer was observed as well as successful distinction between individuals with or without lung cancer overall (23, 24).

**Figure 1.**
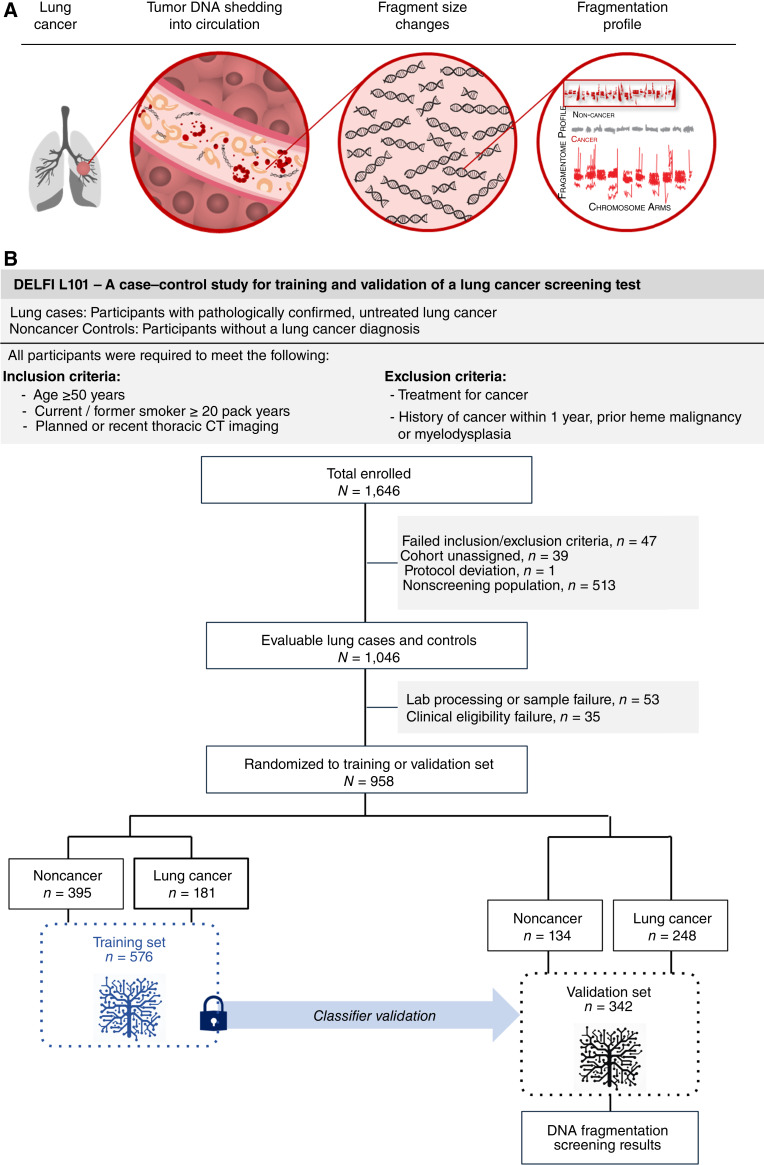
Overall approach to clinical validation of a cell-free DNA fragmentome assay for augmentation of lung cancer early detection. **A,** Illustration representing the DELFI approach for lung cancer through noninvasive assessment of cell-free DNA fragmentation profiles (ratio of short to long cfDNA fragments). Nucleosomal DNA with variable length of linker DNA is released by dying lung cancer cells into the circulation. Genome-wide mapping of the cfDNA fragments demonstrates more aberrant profiles with cancer cell cfDNA fragments compared with the cfDNA in noncancer individuals. **B,** The DNA Evaluation of Fragments for Early Interception–Lung Cancer Training Study, DELFI-L101 study was a prospective case–control study (NCT04825834, including two institutional supplementary protocols NCT00301119 and NCT01775072). The flow diagram illustrates the inclusion and exclusion of L101 participants based on clinical, sample, and assay eligibility criteria and the assignment of evaluable participants to the classifier training (*n* = 576) and clinical validation (*n* = 382) sets. Machine learning of genome-wide cfDNA fragmentation profiles from the training set was used to develop a locked classifier that was evaluated in the clinical validation set.

The fragmentome approach takes advantage of the fact that changes to genomic architecture in cancer cells result in abnormal genome-wide patterns of cell-free DNA in circulation (25). Fragmentation of cfDNA in peripheral blood is nonrandom and reflective of the specific chromatin configurations of the cells and tissues of origin (26). In chromatin-dense regions of the genome, DNA is tightly wrapped around densely spaced nucleosomes and these regions are less accessible to DNA cleavage at the time of cell death through restriction endonucleases and other processes. For regions where the DNA is less tightly packed and spacing between nucleosomes is greater, the DNA is more accessible and, as these cells die, becomes highly degraded. DNA released in the circulation in patients with cancer tends to be more degraded and can be used to identify changes in nucleosomes (27–29). Whole-genome sequencing of cfDNA revealed that the amount and size of cfDNA fragments vary across the genome, depending on changes in chromatin structure (25) and chromosome content (30), leading to the development of the DELFI (DNA Evaluation of Fragments for Early Interception) approach (Fig. 1A; ref. 25). DELFI uses machine learning analyses of whole-genome cfDNA fragmentation profiles to detect cancer and to infer its likely tissue of origin (25).

To convert this approach into a clinically validated cancer detection test, we conducted a multicenter, prospective case–control study, DELFI–Lung Cancer Training Study (DELFI-L101 NCT04825834). Enrollment was inclusive of subjects who met eligibility for lung screening as endorsed by the 2021 USPSTF guidelines (8), which recommends lung screening in individuals between age 50 and 80 who have accumulated 20 pack-years of smoking or more (a pack-year is a product of the number of years an individual has smoked multiplied by the number of packs of cigarettes smoked per day on average during that time span) for individuals who currently smoke of have quit within the past 15 years.

The primary objective of the study was to train and validate a cfDNA classifier for lung cancer early detection that could form the basis of an affordable, high-performing blood-based lung cancer screening test for the current USPSTF screen-eligible population. We used a split-sample approach, in which one “split” was used for training, and the other “split” was used for independent clinical validation. We analyzed fragmentome profiles of the patients with lung cancer in the training set and established their connection to underlying chromosomal and chromatin characteristics. After locking a classifier model based on the training set from the study, we assessed the performance in the validation set. We then modeled the potential population health benefits that could be achieved with the validated test if used even at modest rates to augment current LDCT screening in the United States.

## Results

### Study Design and Subject Characteristics

Enrollment for the L101 study began in March 2021 in 47 centers across 23 states in the United States (Fig. 1B). A total of 958 lung cancer and noncancer control participants were used in the development of the lung cancer screening test in classifier training and clinical validation (Table 1). Roughly three-fifths of subjects contributed to the training set used to develop the classifier, with the remaining two-fifths put aside for the validation set to assess the performance of the classifier once locked. A different share of cases relative to controls was apportioned to each split in order to preserve adequate statistical power for performance estimation in the validation phase of the study.

**Table 1.** Participant demographics and clinical characteristics.

Characteristics, median (IQR); *n* (%)	L101 Study		NHIS 2015^a^	NLST, Both Arms^b^	Silvestri and colleagues (15)
	Training (*n* = 576)	Clinical Validation (*n* = 382)	(*n* = 2,261)(weighted *n* = 13,975,210)	(*n* = 53,452)	(*n* = 1,052,591; USPSTF 2013)
Age (years), median (Q1, Q3)	66 (60, 72)	67 (61, 74)	60 (55, 67)	60.0 (57, 65)	—
Age, *N* (%)					
Age <65 years	243 (42%)	140 (37%)	9,318,186 (67%)	39,234 (73%)	504,794 (53%)^c^
Age ≥65 years	333 (58%)	242 (63%)	4,657,024 (33%)	14,218 (27%)	447,797 (47%)^c^
Sex, *N* (%)					
Male	304 (53%)	191 (50%)	7,788,381 (56%)	31,530 (59%)	544,482 (52%)^c^
Female	272 (47%)	191 (50%)	6,186,829 (44%)	21,922 (41%)	505,318 (48%)^c^
Race, *N* (%)					
White	482 (84%)	328 (86%)	12,313,537 (88%)	48,549 (91%)	461,593 (92%)^c^
Black or African American	47 (8%)	43 (11%)	1,065,711 (8%)	2,376 (4%)	37,111 (7%)^c^
Other^d^	47 (2%)	11 (3%)	595,962 (4%)	2,527 (5%)	5,043 (1%)^c^
Ethnicity, *N* (%)					
Not Hispanic or Latino	422 (73%)	312 (82%)	13,392,848 (96%)	52,118 (97%)	400,737 (98%)^c^
Hispanic or Latino	117 (20%)	40 (10%)	582,362 (4.2%)	935 (2%)	8,807 (2%)^c^
Unknown or not reported	37 (6%)	30 (8%)	0 (0%)	399 (1%)	−(0%)^c^
Education, *N* (%)					
Less than high school graduate	38 (7%, 12%)^c^	20 (5%, 11%)^c^	851,379 (6%)	3,249 (6%)	10,743 (13%)^c^
High school graduate	107 (19%, 33%)^c^	59 (15%, 34%)^c^	1,279,954 (9%)	12,712 (24%)	34,150 (41%)^c^
Associate degree or some college or training after high school	97 (17%, 30%)^c^	45 (12%, 26%)^c^	5,364,443 (38%)	19,711 (37%)	24,758 (30%)^c^
College graduate	57 (10%, 18%)^c^	33 (9%, 19%)^c^	4,425,167 (32%)	8,946 (17%)	13,613 (16%)^c^
Postgraduate or professional degree	25 (4%, 8%)^c^	17 (4%, 10%)^c^	2,027,572 (15%)	7,600 (14%)	
Unknown or not reported	252 (44%, 0%)^c^	208 (54%, 0%)^c^	26,695 (<1%)	1,234 (2%)	−(0%)^c^
Geographic region					
Northeast	143 (25%)	115 (30%)	2,429,248 (17%)	8,713 (16%)	264,819 (25%)
Midwest	135 (23%)	94 (25%)	3,665,934 (26%)	20,953 (39%)	315,702 (30%)
South	207 (36%)	110 (29%)	5,517,263 (39%)	12,775 (24%)	363,509 (35%)
West	91 (16%)	63 (16%)	2,362,765 (17%)	11,011 (21%)	107,502 (10%)
BMI, median (Q1, Q3)	28.1 (24.7, 32.0)	27.6 (23.7, 32.0)	27.4 (23.7, 31.3)	27.3 (24.4, 30.5)	—
Lung cancer USPSTF 2021 criteria, *N* (%)					
USPSTF (2021)	495 (86%)	303 (79%)	13,975,210 (100%)	52,834 (99%)	1,052,591 (100%)
Non-USPSTF (2021)	74 (13%)	60 (16%)	0 (0%)	618 (1%)	0 (0%)
Unknown or not reported	7 (1%)	19 (5%)	0 (0%)	0 (0%)	0 (0%)
Pack-years, median (Q1, Q3)	40 (30, 55)	43 (30, 60)	38 (28, 50)	48 (39, 66)	—
Smoking cigarettes per day, median (Q1, Q3)	20 (20, 30)	20 (20, 30)	20 (15, 20)	25 (20, 35)	—
Years since smoking cessation, median (Q1, Q3)	7.8 (2, 14)	8 (2, 15)	6.0 (3.0, 10.0)	7.0 (3.0, 11.0)	—
Smoking status, *N* (%)					
Current	242 (42%)	168 (44%)	8,178,059 (59%)	25,760 (48%)	645,875 (61%)
Former	334 (58%)	214 (56%)	5,797,151 (41%)	27,692 (52%)	406,700 (39%)
COPD status, *N* (%)					
COPD	264 (46%)	183 (48%)	2,600,068 (19%)	2,690 (5%)	—
No COPD	300 (52%)	189 (49%)	11,365,635 (81%)	50,426 (94%)	—
Unknown or not reported	12 (2%)	10 (3%)	9,507 (<0.1%)	336 (1%)	—
Type II diabetes, *N* (%)					
Yes	124 (22%)	93 (24%)	2,689,713 (19%)	5,174 (9.7%)	—
No	444 (77%)	279 (73%)	10,929,523 (78%)	48,047 (90%)	—
Unknown or not reported	8 (1%)	10 (3%)	355,974 (3%)	231 (<1%)	—
Prior history of Cancer					
Yes	84 (15%)	77 (20%)	2,184,948 (16%)	2,289 (4%)	—
No	492 (85%)	305 (80%)	11,762,865 (84%)	50,951 (96%)	—
Unknown or not reported	0 (0%)	0 (0%)	27,397 (<1%)	212 (<1%)	—

a Subset to LCS-eligible survey respondents as per 2021 USPSTF guidelines, including individuals who had quit smoking; those with a history of lung cancer (unweighted *n* = 45) were excluded.

b NLST demographics, except Geographic region, were calculated internally using NLST data. The numbers are comparable to published estimates (https://www.ncbi.nlm.nih.gov/pmc/articles/PMC2994863/).

c Report percent (%) among those with a reported value.

d Data were aggregated when cell counts were <11 to avoid the risk of reidentification. Other includes Asian, American Indian, Alaskan Native, Native Hawaiian, Other Pacific Islander, Other, Unknown, or not reported.

USPSTF 2013 criteria for lung cancer screening: Age 55–80 years, pack-years ≥30 years, and quit-years ≤ 15 years.

USPSTF 2021 criteria for lung cancer screening: Age 50–80 years, pack-years ≥20 years, and quit-years ≤ 15 years.

With the exception of L101 subjects being somewhat older, the characteristics of enrolled subjects generally paralleled those of the population that is eligible for LDCT screening in the United States as benchmarked against the 2015 National Health Interview Survey (NHIS Data, Questionnaires and Related Documentation, RRID:SCR_025162; Table 1). The similarities are particularly salient with regard to the racial and ethnic representativeness of the L101 subjects. For reference, we also juxtapose the distribution of subject characteristics for enrollees in the National Cancer Institute’s pivotal National Lung Screening Trial (NLST; refs. 4, 31). Enrollment in the NLST was limited to minimums of age 55 and 30 pack-years and exclusion for prior history of cancer within 5 years. L101 subjects were generally more representative of today’s screening eligibility on all fronts, including noncancer control subjects with recent cancer history (with no treatment within 1 year of enrollment)—a group routinely encountered in lung screening programs (32). Demographics of cases and controls in L101 also align with the reported distribution in published screening cohorts (Supplementary Table S1). The distribution of lung cancer histologies and stages among the study’s cases was similar to those observed in LDCT screening studies (Supplementary Table S2; refs. 4, 15). To align with the proposed use of the test, early-stage cancers (Stage I) were substantially overrepresented in the training and validation sets relative to the share of such cancers encountered in incident populations (2).

### cfDNA Fragmentation Profiles of Study Participants

**Figure 2.**
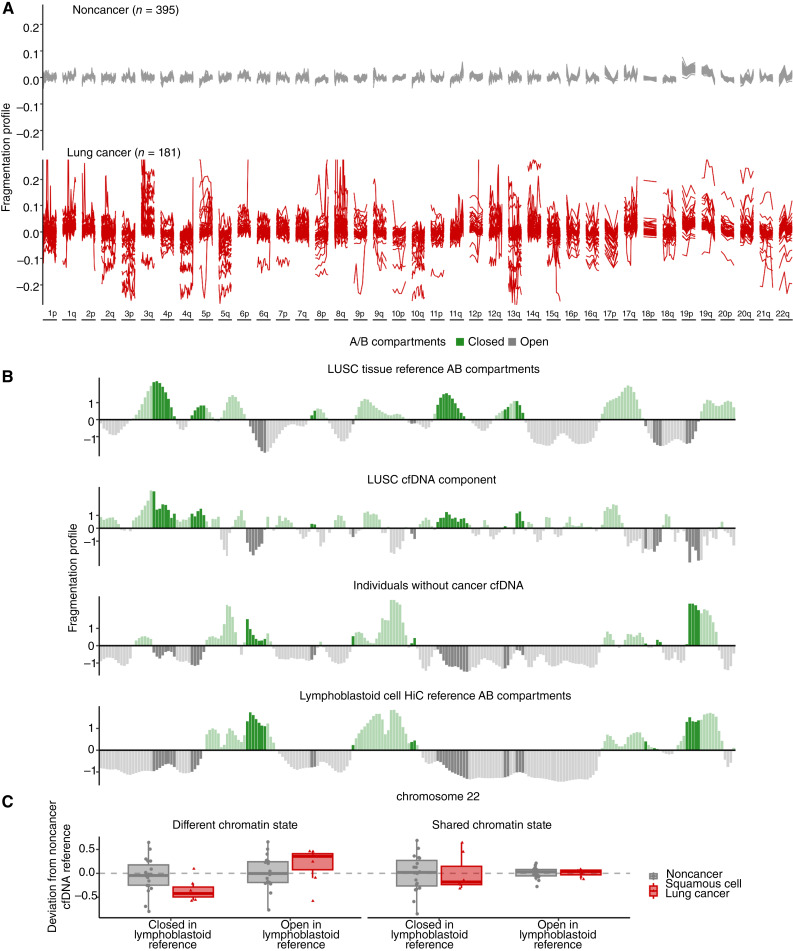
Genome-wide fragmentation profiles are altered in patients with cancer and reflect underlying chromatin structure. **A,** The fragmentation profile (ratio of short to long cfDNA fragments in 5 Mb bins) across the genome was evaluated in the classifier training plasma samples of lung cancer (*n* = 181) and noncancer individuals (*n* = 395). The noncancer individuals had similar fragmentation profiles, whereas patients with lung cancer exhibited significant variation. **B,** Comparison of cfDNA profiles with Hi-C A/B chromatin compartment reference data from lung cancer tissue or peripheral blood cells. Track 1 shows A/B compartments extracted from LUSC cancer tissue (48). Track 2 shows a median lung cancer component extracted from the LUSC plasma samples of 7 patients with lung cancer from the classifier training set with high tumor fraction by ichorCNA (49). The 7 LUSC cases with high ichorCNA have values of 0.051, 0.439, 0.230, 0.259 0.439, 0.167, and 0.057. Track 3 shows the median profile for 10 noncancer plasma samples from the training set. Track 4 shows A/B compartments for lymphoblast cells (48). These four tracks show chromosome 22 as an example, with darker shading indicating informative regions of the genome where the two reference tracks differ in domain (open/closed). **C,** 100-kb regions were selected using the reference LUSC and lymphoblast A/B tracks as having the same chromatin state or opposite chromatin state. Within these regions, the deviation of the fragmentation value from a noncancer cfDNA reference (*n* = 10) was plotted per region per individual (noncancer *n* = 20, LUSC *n* = 7). Values around 0 have little variation from the noncancer reference. Negative values indicate a region has a more open chromatin state than the reference and positive values indicate a region has a more closed chromatin state than the reference. These data suggest that although cfDNA profiles of healthy individuals reflect the chromatin structure of blood cells, those of patients with lung cancer represent a mixture of cfDNA patterns of chromatin compartments from lung cancer as well as blood cells.

Fragmentation profiles provide a means of visualizing variations in features of the cfDNA fragmentome, including fragment length and sequence coverage, across the genome within and across study populations. We examined low coverage (∼3×) whole-genome sequence data from our training set to evaluate fragmentation profiles in 504 nonoverlapping 5 MB regions with high mappability, each region comprising ∼80,000 fragments, and spanning 2.5 GB of the genome. We observed genome-wide consistency of fragmentation profiles across noncancer control subjects (*n* = 395) from the training set in the L101 study (Fig. 2A). This consistency was similar to our previous observations of the cfDNA fragmentomes among individuals without cancer (23, 25). Case subjects with a lung cancer diagnosis (*n* = 181), by contrast, displayed extensive genome-wide variation.

To understand the origins of the cfDNA fragmentation patterns, we compared these with chromosome conformation capture (Hi-C) open (A compartment) and closed (B compartment) chromatin (Fig. 2B). Analysis of cfDNA profiles from 7 patients with lung squamous cell carcinoma (LUSC) revealed that their fragmentomes reflected two components. One resembled cfDNA profiles of individuals without cancer and another with high similarity to A/B compartments previously estimated from LUSC tissues. In contrast, the cfDNA patterns of individuals without cancer resembled the Hi-C data of lymphoblastoid cells. To further quantify this phenomenon, we extracted regions where the chromatin state was shared between the LUSC A/B reference track and the lymphoblastoid A/B reference track as well as regions where the chromatin state was different between the two tissues. Within the shared regions, the fragmentation of samples from individuals with or without cancer was nearly identical (Fig. 2C). In the altered chromatin regions, the deviation of fragmentation from the healthy reference for the patients with cancer was shifted, indicating more closed chromatin in cancer cfDNA in regions of closed LUSC compartments, and the opposite effect for closed regions in the lymphoblastoid reference. These analyses suggest that cfDNA fragmentation from individuals with lung cancer represents a mixture of cfDNA profiles of chromatin compartments of cells from peripheral blood as well as those from lung cancer.

**Figure 3.**
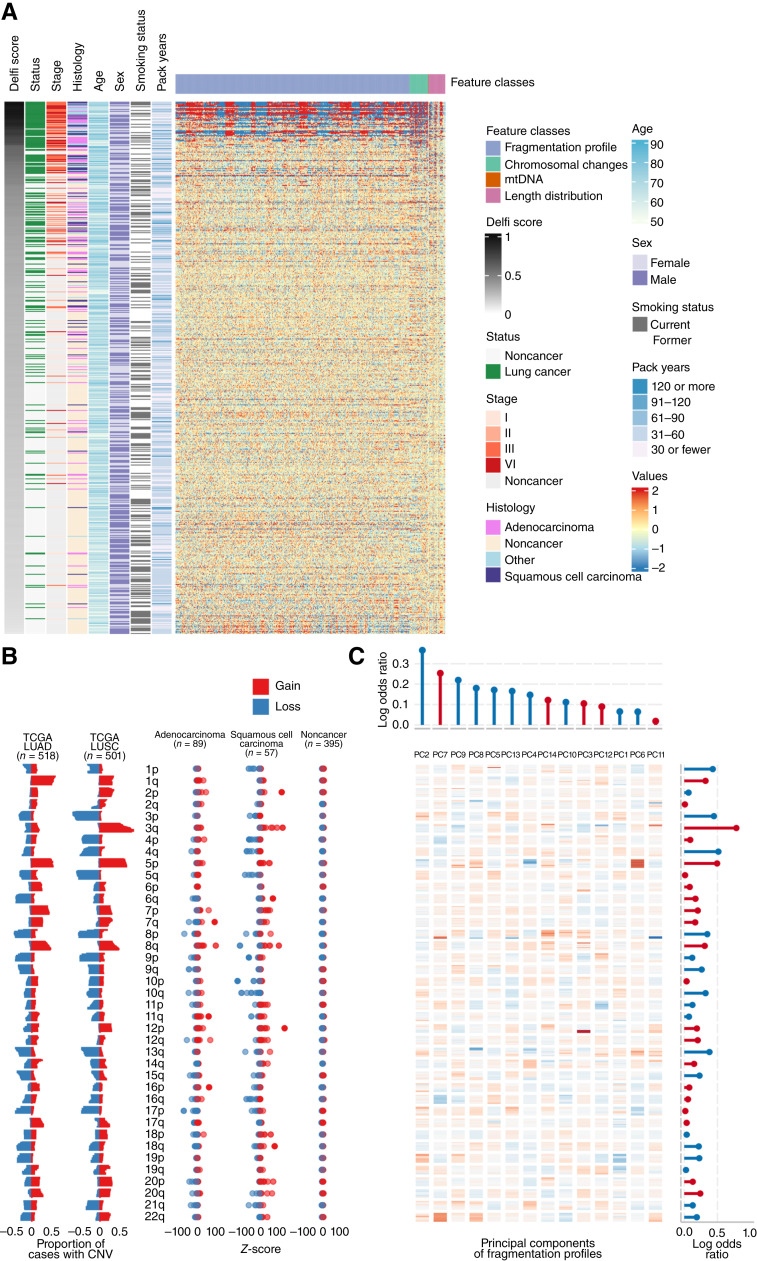
High-dimensional fragmentation features reflect lung cancer biology and are incorporated in the machine learning classifier. **A,** Heatmap representation of the deviation of cfDNA fragmentation features across the genome for the classifier training set with lung cancer or noncancer individuals compared with the mean of classifier training noncancer individuals. Each row represents a sample, whereas columns show individual genomic features. The cross-validated DELFI score and clinical characteristics are indicated to the left of the fragmentation deviation heatmap. **B,** Left, TCGA-derived observations of chromosomal arm gains (red) and losses (blue) in lung adenocarcinoma (LUAD; *n* = 518) and squamous cell cancer tissues (LUSC; *n* = 501). Right, the observed chromosome arm gains (red) and losses (blue) in the classifier training individuals separated by histology. **C,** A heatmap representation of the principal component eigenvectors of the fragmentation profile features. Regression coefficients from the final classifier indicating how the principal components of the fragmentation profiles and *z*-scores of the chromosomal arms were combined are provided in the top and right margins of the heatmap, respectively. Positive values for the coefficients are represented in red, whereas negative values are represented in blue. Agreement across copy number chromosomal gains and losses in TCGA lung cancers, observed *z*-scores in the cfDNA of patients with lung cancer, and chromosome arm model coefficients reflect biologic consistency between chromosomal changes in lung cancer, cfDNA fragmentation profiles, and classifier features.

As the cfDNA fragmentome may reflect large-scale genomic alterations within cancer cells, we assessed cfDNA chromosomal gains and losses in the circulation of these patients. In addition to the diverse feature set captured by genome-wide fragmentation profiles resulting from chromatin changes (Fig. 3A), our analyses revealed changes in the representation of chromosomal arms commonly gained or lost in lung cancer as reported previously in The Cancer Genome Atlas (TCGA) large-scale analyses (lung adenocarcinoma, *n* = 518; LUSC, *n* = 501; Fig. 3B). Subjects with lung adenocarcinoma or LUSC cancer show increased cfDNA representation of 1q, 3q, 5p, 8q, and 12p and decreased levels of 1p, 3p, 4q, 5q, 10q and 17p, all known to be gained or lost in these malignancies (Fig. 3B; refs. 33, 34).

We used the combination of these distinct features, the relative contribution of which is captured through machine learning, to examine differences between cancer and noncancer subjects. For some advanced cancers, single genomic features seemed sufficient for their identification. No appreciable confounding by subject characteristics such as smoking status, demographic, or clinical characteristics, was observed (Fig. 3A). Principal component analysis was employed to derive linear combinations of fragmentation features that explained at least 95% of the variance. To develop a classifier to detect differences between individuals with or without cancer, we incorporated the resulting components, chromosomal arm level changes, fraction of cfDNA derived from the mitochondrial genome, and the overall distribution of cfDNA fragment lengths into a penalized logistic regression machine learning model (Fig. 3C). In combination, the classifier generated a continuous score with range 0 to 1 that represented the estimated probability of case rather than control status.

### Classification and Cross-Validated Performance

Converting the classifier’s continuous score (0–1) to a qualitative binary “positive” or “negative” result involves choosing a “cutpoint.” That choice is informed by the clinical context for the test. For a blood test developed to increase the detection of lung cancer in a population that is eligible for LDCT screening, test sensitivity—identifying cancer when it is present—is the priority. The preference for high sensitivity can require some sacrifice in test specificity, which is the same as accepting some increase in the false positive rate. The tradeoff is acceptable in this clinical context because in the population to be tested, LDCT screening is currently recommended as a primary screen, and a positive blood test (whether true or false positive) would be followed by that same recommended LDCT evaluation.

Sensitivity of less than 100% would still imply some false negative test results, meaning the blood test would miss some individuals who have lung cancer. The false negative rate should be considered in two contexts. First, the clinical use of the blood test is to improve uptake in those not having LDCT screening (in whom 100% of lung cancers fail to be screen-detected today). Second, the acceptable rate of missed cancers is a function of how common they are among the test negatives, as prevalence is a primary driver of the risk-to-benefit tradeoff of screening. Thus, target performance in clinical validation was established to be 80% sensitivity across all stages, weighted for the stage distribution seen in lung cancer screening, which would result in a prevalence of lung cancer in the test negatives of less than 0.3%.

At the selected cutpoint of 0.22, tenfold cross-validation with 10 repeats within the training population resulted in stage-weighted overall sensitivity of 84% (95% CI, 78%–90%) with sensitivity of 75% for stage I (median score = 0.26), 90% for stage II (median score = 0.39), 96% for stage III (median score = 0.53), 97% for Stage IV (median score = 0.61) and specificity of 50% (Supplementary Fig. S1). The cross-validated estimates, which were generated internally for the training set, provided a strong indication that the approach would meet the 80% sensitivity target in clinical validation.

### Clinical Validation and Contextualization of Test Performance

**Figure 4.**
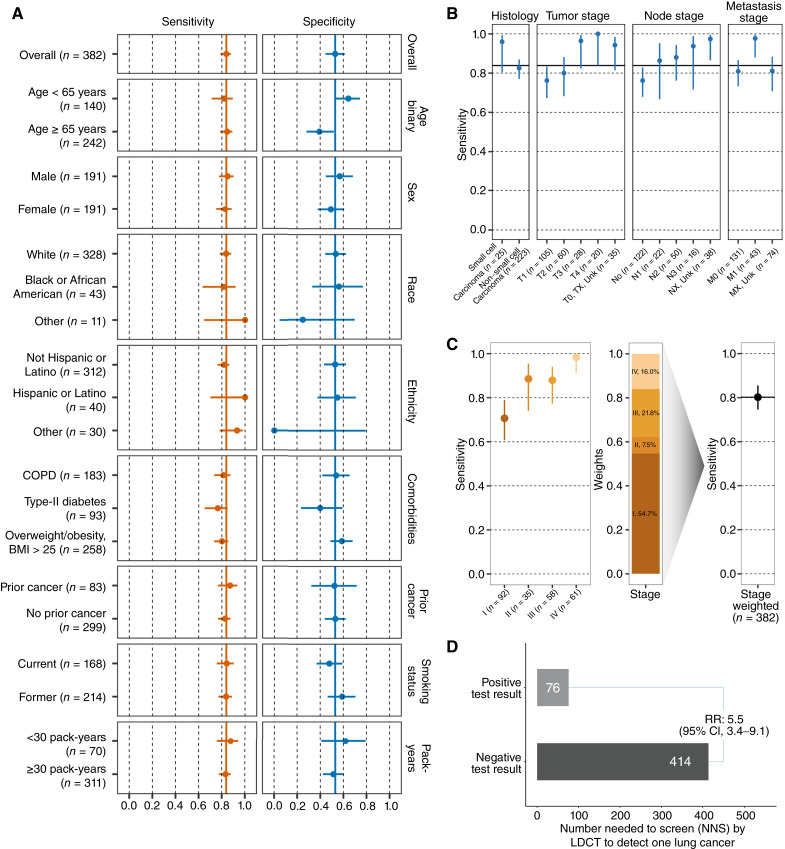
Performance of blood-based lung cancer screening test. **A,** Sensitivity and specificity of the test in the clinical validation set (*N* = 382) overall and by clinical subgroup. Point estimates are reported with 95% Wilson confidence intervals. Overall sensitivity and specificity denoted by solid vertical lines. **B,** Sensitivity of the test in the lung cancer cases in the clinical validation set (*N* = 248) evaluated across cancer histology, and T, N, and M categories. Point estimates are reported with 95% Wilson confidence intervals. Overall sensitivity of 84% denoted by the solid horizontal line. **C,** Left, sensitivity of the test in the lung cancer cases in the clinical validation set (*N* = 246) by cancer group stage. Middle, bar plot showing the stage distribution of lung cancer as observed in populations undergoing lung cancer screening with LDCT (based on NLST study) that are used to weigh observed stage-specific sensitivities. Right, lung cancer screening relevant stage-weighted sensitivity in clinical validation set. **D,** Comparison of the NNS with LDCT conditioned on test positive or negative result when applied in the lung cancer screening eligible population. Test performance showed consistency across clinical subgroups and expected increased performance with increasing burden of disease (tumor (T), node (N), metastasis (M) and group staging). After weighting, the stage distribution to reflect a screening population, test performance remained high and demonstrated the ability to reliably identify those individuals more likely to have lung cancer detected on LDCT.

The locked classifier and cutpoint were then assessed in the L101 clinical validation subjects, comprising 248 individuals with cancer and 134 without cancer. Observed sensitivity was 84% (95% CI, 79%–88%), and observed specificity was 53% (95% CI, 45%–61%; Fig. 4A; Supplementary Fig. S2A and S2B). Test performance was robust across subgroups defined by patient categorical sex, race, ethnicity, presence of comorbidities, history of prior cancer, and smoking status (Fig. 4A). Sensitivity was consistent across age groupings. As has been observed with other biomarker cancer detection tests, specificity was greater in younger individuals (35, 36). Performance in individuals with chronic obstructive pulmonary disease [sensitivity of 81% (95% CI, 73%–87%), specificity of 54% (95% CI, 42%–65%)], a frequent comorbid condition in populations eligible for lung cancer screening, was similar to the overall group. There was no evidence that the presence of noncancerous lung nodules among controls reduced test specificity (Supplementary Table S3).

Among the cancer cases in the clinical validation set (*N* = 248), sensitivity was consistent across categories of disease and patient groupings. It was increased in patients with small cell lung cancer compared with those with nonsmall cell lung cancer cell type, similar to prior reports (Fig. 4B; ref. 24). It also rose across T and N stages and in the presence rather than the absence of metastases (Fig. 4B). Sensitivity also increased in association with overall stage (Fig. 4C; *N* = 246 due to exclusion of 2 participants with unknown group stage): Stage I = 71% (95% CI, 61%–79%), Stage II = 89% (95% CI, 74%–95%), Stage III = 88% (95% CI, 77%–94%), and Stage IV = 98% (95% CI, 91%–100%).

Test sensitivity and specificity should be contextualized to the population in which testing is planned, and that population may not match the one enrolled in a case–control study. We obtained the screening population distributions for lung cancers from the LDCT arm of the NLST, and the age distribution for nonlung cancers from the 2015 NHIS, a national survey that is routinely used to characterize the population eligible for lung cancer screening in the United States (4, 37, 38). The resulting screening population sensitivity and specificity were 80% (95% CI, 75%–86%; Fig. 4C) and 58% (95% CI, 49%–66%).

Test performance should also be contextualized with regard to the expected disease frequency in the intended use population. For lung cancer screening, the projected detectable lung cancer prevalence is 0.7% (39, 40). At this prevalence, the negative predictive value (NPV) of the validated test is 99.8%, and the positive predictive value (PPV) is 1.3%. These statistics contrast to the unselected probability of lung cancer being found (a PPV of 0.7%) or not found (an NPV of 99.3%) based on screening eligibility alone. In a population health context, it is common to characterize these statistics in terms of the “number needed to screen” (NNS) with an LDCT scan to detect one lung cancer. In the screening eligible population, the NNS is 143. Based on the validated test performance, positive and negative test results are associated with NNS with an LDCT to find a lung cancer of 76 and 414, respectively—a relative risk of 5.5 (95% CI, 3.4–9.1; Fig. 4D).

### Potential Population Health Outcome Improvements through Test Implementation

**Figure 5.**
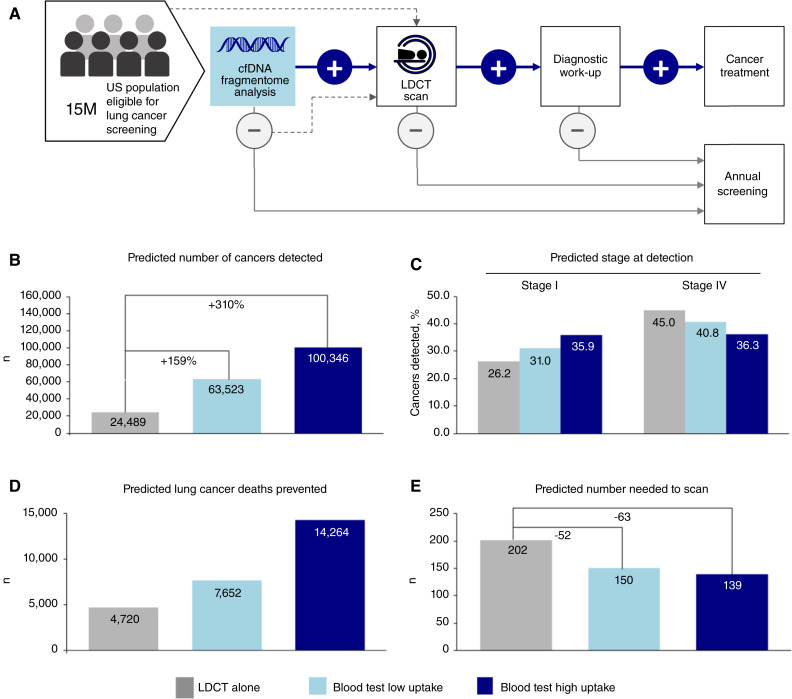
Population health benefits of a blood-based test in lung cancer screening. **A,** Care pathway reflecting the recommended standard of care for lung cancer screening with LDCT that is received by 6%–10% of eligible individuals annually, as well as potential pathway employing initial blood-based test and follow-on events. **B,** The predicted number of cancers detected by screening scenario: LDCT alone (“base case”); LDCT + low test uptake; LDCT + high test uptake. **C,** Predicted cancers diagnosed at stage I *versus* Stage IV by screening scenario: LDCT alone (“base case”); LDCT + low test uptake; LDCT + high test uptake. **D,** Predicted decrease in lung cancer deaths represented by screening scenario: LDCT alone (“base case”); LDCT + low test uptake; LDCT + high test uptake. **E,** Simulated comparison of the predicted number needed to scan with LDCT to detect one lung cancer: LDCT alone (“base case”); LDCT + low test uptake; LDCT + high test uptake. Population-level modeling demonstrates significant health benefits when a blood-based test is available as an alternative for lung cancer screening.

We examined how differing hypothetical rates of uptake of the test, which we developed with the express purpose of improving overall lung cancer screening rates, could potentially improve population-level outcomes (see Supplementary Methods for details). The 5 years modeled scenario (Fig. 5A), in which we used the performance of the test documented in clinical validation, contemplates that there is a share of individuals who continue to receive primary LDCT screening, whereas some share of the remaining (far larger) population would be evaluated with the new test. The Monte Carlo simulation involved a single population of 15 million individuals eligible for lung cancer screening, a number that parallels the number of eligible individuals in the United States today (Fig. 5A; ref. 40).

Three scenarios were considered: A “base” case, reflecting current practice in the absence of a blood–based screening test in which 6% of eligible individuals were screened with LDCT in the 1^st^ year, and over the 5-year time period the percentage screened with LDCT rose to 9%. This was compared with two additional scenarios, in which either 10% (“low” scenario) or 20% (“high” scenario) of individuals who are eligible for lung cancer screening but not receiving LDCT had the blood-based test in the 1^.^ year, rising in a linear fashion to 25% and 50%, respectively, by year 5. In real-world applications, the new test result would be incorporated into a shared decision-making visit. In order to consider the impact of patient behavior, we modeled the rate of follow-on LDCT evaluation as 80% (for blood test positives) or 10% (for blood test negatives; refs. 41, 42). Individuals could not move between the two alternative screening approaches between years.

Compared with the hypothetical “base” case, each of the “low” uptake and “high” uptake blood test adoption scenarios resulted in multiple population-level outcome changes. Comparing the low and high scenario to the base case, respectively, the number of lung cancers detected by screening rose from 24,489 to 63,523 and 100,346 (Fig. 5B), the proportion of stage I disease diagnoses rose by an absolute 4.8% (from 26.2% to 31.0%) and 9.7% (from 26.2% to 35.9%; Fig. 5C), and Stage IV lung cancer diagnoses fell by 4.2% and 8.7% (Fig. 5C). In terms of prevented lung cancer deaths, there were 4,720 prevented in the base case, 7,652 with low uptake of the blood test, and 14,264 in the high uptake scenario (Fig. 5D).

When incorporated as part of screening, the likelihood that LDCT detects cancer increases, with the NNS with LDCT to detect a single lung cancer decreasing from 202 in the base case to 150 and 139 in the “low” and “high” scenarios (Fig. 5E). Results generated with alternative modeling assumptions are included in Supplementary Table S4, in which across all scenarios there is general improvement in all analyzed outcomes. In a scenario in which there is a 50% probability of follow-up LDCT following either a positive or negative blood test result (making the LDCT random with respect to the test result), there are improvements in lung cancer outcomes, but the NNS with LDCT to find a lung cancer is essentially unaltered (base case NNS = 202, low uptake case NNS = 201, high uptake case NNS = 204; Supplementary Table S4). Overall, these simulations highlight the potential benefit of our blood test for improving lung cancer outcomes and increasing the efficiency of LDCT screening.

## Discussion

More than 13 years have passed since November 2010 when the initial positive findings of the NLST were announced (4). Guideline endorsement and insurance coverage for annual LDCT screening in individuals at elevated risk due to their age and smoking history followed soon thereafter in the United States. At present, despite lung cancer being the number one cause of cancer death in men and women in the United States, lung cancer screening rates are meager, with reported annual rates of 6% to 10% (1, 8, 10, 11). Even among those who undergo lung cancer screening, the frequency with which people come back next year for an annual screen are reportedly in the range of 20% to 50%, far lower than rates of screening for other recommended cancers (14). The potential public health benefits of increasing lung cancer screening rates are sizable.

One means of improving rates is to expand the menu of screening test choices for patients (13, 18, 41, 42). The archetype of this approach is colorectal cancer screening. The USPSTF endorses multiple differentiated alternatives to colonoscopy (recognized as the gold standard test). Stool-based tests, such as the recommended FIT and gFOBT tests, can be performed at home. CT (“virtual”) colonoscopy is a radiologic examination. Flexible sigmoidoscopy requires neither anesthesia nor an overnight bowel cleansing regimen. Even though the sensitivity of these tests in some cases decreases well below the sensitivity of colonoscopy (FIT has pooled sensitivity for colorectal cancer of 74%), the Task Force’s recommendation reflects the public health priority of achieving high screening rates (43).

A blood-based test for the early detection of lung cancer could augment rates of lung screening in a similar fashion, by serving as an initial test for individuals who are eligible but not receiving LDCT. A positive blood test would then “reflex” to a screening LDCT, in much the same way that a positive FIT or gFOBT test reflexes to a screening colonoscopy (a schematic of this clinical path is shown in Fig. 5A). If the screening LDCT is then negative for lung cancer (in the United States, this is equivalent to having a Lung-RADS 1 or 2 result), the blood test would be considered a false positive and the patient would merely return in a year for another evaluation (44). In other words, the blood test result informs the decision regarding if a subsequent screening LDCT is more or less likely to discover a lung cancer, but should not influence the management of that LDCT’s findings or be considered an input while assessing the likelihood of a particular LDCT detected nodule is or is not cancerous.

Efforts have been ongoing to develop a biomarker-based early detection test for lung cancer, but no clinically validated biomarker tests have demonstrated the requisite sensitivity for disease detection that is required for a test to augment recommended screening (20–22). An autoantibody-based test called EarlyCDT demonstrated 41% sensitivity for lung cancer when used in clinical practice (45). When weighted for the lung cancer stage distribution observed in LDCT screening, the methylation-based Galleri test had a reported 53% sensitivity for lung cancer in a case–control study enrolling a general population of patients rather than those selected for lung cancer screening eligibility (36). In contrast, proof-of-concept analyses of cfDNA fragmentomes, assessed via low-coverage whole-genome sequencing that detects millions of cfDNA fragments genome-wide, have demonstrated high rates of cancer detection including for early-stage disease, likely due to the ability to identify signals resulting from widespread genomic and chromatin abnormalities of cancer cells (23, 25). Although the blood-based test examined in this study focused on these previously described fragmentome features (23, 25), it is conceivable that it could be augmented in the future with additional characteristics that are detectable through genome-wide cfDNA analyses (24).

We conducted a prospective case–control study (DELFI-L101) with the aim of developing a high-sensitivity lung cancer early detection test that could improve lung cancer screening utilization. Nearly 1,000 case and control subjects from the intended use lung cancer screening population allowed us to train, lock, and independently validate a test that for the intended use population has 80% sensitivity and 58% specificity. Performance of the test was consistent across patient demographics, which generally paralleled the screen-eligible population, lung cancer cell types, and clinical characteristics. Sensitivity was strong across stages of lung cancer.

Our test specificity of 58% must be understood within the context of the test’s clinical use. Although it is true that this specificity equates to 42% of individuals who do not have lung cancer receiving a “false positive” test result, the reason false positives are a potential concern is because of what they lead to in terms of health care interventions, costs, and patient experience. That our test is designed for and specifically focused on the population that is currently recommended to undergo LDCT screening clarifies the implications of the false positive rate. The only clinical step that should follow a positive blood test result is an LDCT. This stands in contrast to the implications of a false positive result for screening tests that identify multiple cancers in broad populations, for which the follow-up evaluation may not be tightly defined.

When applied to the lung cancer screening eligible population in which the prevalence of lung cancer is 0.7%, the resulting numbers needed to screen with LDCT to identify one lung cancer associated with positive and negative results are meaningfully distinct, and as such, could help guide the shared decision-making that patients and their providers engage in when considering LDCT screening. Defined in this manner, the test’s NNS in association with a positive result is 76, making the LDCT lung cancer discovery rate roughly twice as high if performed on those with a positive blood test result compared with the rate when all those eligible are screened with an LDCT. Put another way, the PPV of the positive blood test (1.3%) is approximately twice that of LDCT eligibility criteria alone (0.7%). A direct consequence of this is that the PPV of LDCT itself is roughly doubled. As such, the benefit-to-risk calculus for LDCT screening is improved by increasing the likelihood cancer is found by LDCT without changing the likelihood a benign nodule is found (i.e., the test’s specificity is consistent whether or not there are benign nodules).

For those with a negative result, the NPV is 99.8%, a summary of the estimate that 414 test-negative individuals would need to be screened with LDCT to find a single case of lung cancer. This low probability, when incorporated into a shared decision-making visit, should aid patients and physicians regarding the decision about a subsequent LDCT scan. Today, lung cancer screening recommendations prioritize individuals who have a higher probability of having detectable disease, primarily because a high probability of lung cancer is associated with screening having an improved benefit-to-risk ratio when the likelihood of disease detection is greater. The risks of LDCT screening are well characterized, and they include the radiation from the CT scan itself, and more troubling, the discovery of abnormalities that are not clinically meaningful but spur further evaluation nevertheless.

To balance these potential harms against the benefit of early lung cancer detection, guideline groups typically select a threshold for the likelihood of lung cancer being present that defines the level above which screening is recommended and below which it is felt to be more harmful than beneficial. That threshold today in the United States is approximately a 0.5% likelihood of lung cancer diagnosis within 1 year, a statistic intrinsic to the screening guidelines published by the USPSTF in 2021 (8) and consistent with the risk thresholds proposed by the American College of Chest Physicians for screening based on several available risk prediction models, and recommendations from other authors (46, 47). The test’s NPV of 99.8% is equivalent to a 0.2% probability of lung cancer being detected by a subsequent LDCT, far lower than the 0.5% threshold proposed in the guidelines.

Our population-level modeling demonstrates the potential a blood-based test with these performance characteristics holds. At even modest rates of adoption, such as 10% rising to 25% within 5 years, meaningful reductions in late-stage diagnoses and deaths from lung cancer would be observed, numbering in the thousands per annum. Improving lung cancer screening utilization would help achieve Healthy People 2030 goals, including increasing the proportion of USPSTF screen-eligible adults who get lung cancer screened to 7.5% and reducing lung cancer mortality rates by 21% (8).

Our study should be understood within the context of its limitations. Although we took great care to enroll a population that was a cross-section of those eligible for lung cancer screening, and achieved demographic and clinical representation reflective of the screening population, our study is still a case–control analysis. Although we were able to contextualize the test performance for the intended use population, the study may still have selection biases that we cannot detect. Similarly, although CT scans were required for study enrollment, some were performed prior to blood collection. This sequence is different from how the test would be applied in clinical use, in which the test would be used to evaluate whether subsequent LDCT is appropriate. Our analyses suggest that the difference in timing did not bias our findings. Lastly, our study enrollment criteria parallel the intended use population of those currently eligible for lung cancer screening in the United States. As such, we have little insight into whether the test would generalize to other populations at risk for lung cancer, such as individuals with occupational risk factors.

Case–control results can be imperfectly predictive of prospective performance—a key reason why we are also conducting cohort studies in the context of lung cancer in the United States (NCT05306288) and Europe (Netherlands Trial Register NL9710) to further evaluate our fragmentome-based classifier. Providing some reassurance on this front, our previous proof-of-concept study was an analysis of a prospective cohort, which also demonstrated high sensitivity across stages and strong discrimination between individuals with and without lung cancer (23). Similarly, although our models show potential population health benefits—including numerous prevented deaths from lung cancer across a range of blood test utilization rates—they do not consider other important outcomes, such as the impact on patient lifespan or quality of life, nor tie costs to those outcomes. Whether the test will be used at these projected rates is a focus of an ongoing study (DELFI L301 NCT06145750).

Concerns have also been raised about the observation that liquid biopsy tests are more sensitive when the cancer that is present is more biologically aggressive—a phenomenon that may be independent of the stage or cell type of the disease (22, 23). If liquid biopsy tests systematically find aggressive diseases, but screening’s benefits are conferred primarily through detection (and treatment) of more indolent diseases, this would raise concerns about assumptions of patient benefit resulting from liquid biopsy detection. In our study, this concern is mitigated on two fronts: our validation was conducted in individuals who were eligible for lung cancer screening and in many cases undergoing it, and in that context, the test sensitivity is high enough (80%) that detection almost certainly overlaps with the cancers that are required for early detection’s benefits (the mortality benefit from LDCT screening is between 20% and 24%).

There is little question that to achieve screening at the population scale, tests must be available at a low cost, something the low-coverage whole-genome multifeature sequencing–based test we assessed enables. Utilizing this affordable approach, to analyze cfDNA fragmentation patterns in individuals with or without lung cancer, we developed and validated a novel blood-based lung cancer screening test that has high performance. Although we await further validation in ongoing prospective cohort studies, modeling suggests substantial public health benefits if a test like this can improve lung cancer screening participation among those who are not currently receiving it.

## Methods

### Patient Selection, Study Design, and Sample Acquisition

We conducted a multisite, prospective, observational, case–control study to train and validate a cfDNA classifier for lung cancer detection using DNA fragmentomes identified through low-pass whole-genome sequencing (DELFI L101 NCT04825834). The DELFI-sponsored L101 study protocol was approved by a central IRB, WCG IRB, and also by the site local IRB if required. Two sites enrolled participants under supplementary institutional protocols approved by their respective institutional IRBs (NYU Lung Cancer Biomarker Center NCT00301119; and MSKCC Lung Cancer Training Study NCT01775072). All participants signed written informed consent and all study-related procedures were conducted in accordance with recognized ethical guidelines (International Ethical Guidelines for Biomedical Research Involving Human Participants, the Declaration of Helsinki, and applicable state and local regulations).

Eligibility criteria were constructed to enroll participants representative of the elevated-risk population eligible for lung cancer screening based on the 2021 USPSTF criteria (8). Participants 50 years and older, who currently or previously smoked, and with a smoking history of 20 pack-years or more were eligible to enroll. Enrollment was allowed for individuals more than age 80 and who had quit smoking for more than 15 years—each is a distinction from the USPSTF lung cancer screening eligibility criteria. These inclusion criteria were applied consistently across all of the enrolling protocols to confirm eligibility for enrollment on L101. Enrollment required a thoracic CT scan within 12 months of enrollment or planned within 6 weeks after. The average time interval was 35 and 46 days for cases and controls, respectively. There was no association (all *P* values > 0.05) between the timing of the CT scan and the enrollment blood draw regarding the test result either among cases or controls. This was the case whether we examined the absolute value of the time difference between the CT scan and the blood draw, examined subjects with CT before the blood draw or the reverse separately, and evaluated the DELFI score as a continuous variable or a binary test result or whether the predictor was continuous time or separated as time intervals more than 60 days or within 60 days.

Individuals were excluded if they had prior treatment for any cancer within 1 year of enrollment, a history of hematologic malignancy or myelodysplasia, organ tissue transplantation, blood product transfusion within 120 days prior to enrollment, and current pregnancy; were enrolled in another DELFI-sponsored study; or had any condition that in the opinion of the investigator should preclude the participant joining the study. These exclusion criteria were consistent across all of the enrolling protocols.

Participants were enrolled under either a central or local IRB-approved protocol at 47 sites in the United States that were diverse geographically and with respect to academic or private institutional structure. Sites with thoracic surgery clinics, nodule follow-up clinics, and large centralized screening programs were selected to maximize enrollment of early-stage cancers representative of a screening population. Participants were enrolled after written informed consent, eligibility confirmation, and blood specimens were collected. No results were returned. Patient demographics, medical history, and other diagnostic procedures and results were abstracted from medical records. Subjects were allocated to Group A (lung cancer), a label that required pathologic confirmation, Group B (noncancer controls), or Group C (cancer other than lung cancer). Participants with a pending resolution of abnormal thoracic CT findings were classified temporarily as Group indeterminate (Supplementary Methods Fig. S2).

For lung cancer cases (Group A), we approached staging in a manner paralleling the approach taken in the NLST. Medical records, pathology, and tumor-staging reports were obtained for all lung cancer cases. Disease stage was determined according to the Cancer Staging Manual of the American Joint Committee on Cancer, based on the best available information at the time of enrollment (pathologic staging if available, otherwise clinical stage).

Medical records were rereviewed 12 months postenrollment to identify interval cancers, resolve indeterminate status, and reclassify individuals when relevant. For subjects without a 12-month medical record review, lung cancer status was based on enrollment classification. Unresolved indeterminates were excluded from training and validation. For the purpose of classifier training and validation, we included samples obtained under the conditions and processes that were anticipated for future screening implementation. For example, Group B was limited to those enrollees whose thoracic CT scan was obtained for the purpose of lung cancer screening, as opposed to a nonscreening, clinical diagnostic indication (see Supplementary Methods).

### Plasma Sample Collection and Sequencing Library Preparation

Plasma collection, cfDNA extraction, genomic library preparation, and next-generation sequencing were performed as described in Mathios and colleagues (23) with the following modifications. Peripheral blood was collected in Streck tubes from each participant within 30 days of enrollment, shipped to a central lab for processing into plasma and buffy coat aliquots, and stored at −80°C for cfDNA analysis. Plasma samples were processed by DELFI Diagnostics using its standard laboratory and bioinformatics protocols, adapted for high throughput automation on eight-channel Microlab STAR and STARLET liquid-handling robots (Hamilton Company; ref. 24). All procedures were performed in a Clinical Laboratory Improvement Amendments–certified laboratory operated by DELFI Diagnostics. Libraries were prepared from extracted DNA with KAPA HyperPrep DNA kits (Roche Molecular Systems, Inc.) and pooled and sequenced on a NovaSeq 6000 system (Illumina, Inc.).

For classifier training, samples had libraries prepared in 12 batches (Supplementary Table S5). Samples were randomly allocated to each batch, maintaining a similar balance of lung cancer and noncancer by batch. In the clinical validation set, samples had libraries prepared in six batches, in which a similar process of randomization was performed.

### Whole-Genome Fragmentation Analyses

FASTQ files were processed via DELFI’s standard bioinformatics pipeline. Reads were trimmed of adapter sequences using fastp and aligned against the hg19 human reference genome using Bowtie 2 with duplicate reads removed by Samtools. Aligned paired end reads were converted to genomic intervals representing the sequenced DNA fragment using bedtools.

To capture large-scale differences in fragmentation across the genome from low-coverage whole-genome sequencing, we tiled the human reference genome into 504 non-overlapping 5 Mb bins. Following the approach previously described (26), fragments were assigned to bins based on their alignment position in the reference genome and were categorized as short (100–150 bp in length) or long fragments (151–220 bp in length). Following a locally weighted scatterplot smoothing (LOESS) for GC correction, the ratio of short fragments to long fragments was calculated for each bin and centered by the autosomal mean ratio for each individual. To reduce the dimensionality of the fragmentation profiles in training, we performed a principal components analysis, retaining the number of principal components explaining 95% of the variation between participants (23). To estimate variation between individuals in the overall distribution of fragment lengths across the genome, we approximated the fragment length distribution by fitting a mixture of gaussian distributions. Chromosome arm changes in overall coverage were measured using *z*-scores and calculated for all 39 acrocentric chromosomal arms as previously described (25). The mitochondrial genome representation and short/long ratio was calculated, as described above, across the mitochondrial genome (∼17 kb).

### Chromatin Structure Analysis

Cell-free DNA fragmentation patterns as shown in Fig. 2B were generated by performing GC correction independently for mononucleosome length reads (100–220 bp) and dinucleosome length reads (260–420 bp) and calculating the ratio of mononucleosome to dinucleosome reads in 100-kb tiled bins across the hg19 reference. The median chromatin profile from 10 participants in the L101 study without cancer was used to obtain a non-cancer reference. A/B compartments for lung cancer tissue and lymphoblastoid cells were obtained from https://github.com/Jfortin1/TCGA_AB_Compartments as well as from https://github.com/Jfortin1/HiC_AB_Compartments as described previously (48). The two reference tracks were compared with identify informative 100-kb bins. Informative bins were found by min–max normalizing the eigenvectors. We defined differentially open bins in the LUSC chromatin track as those with one standard deviation or more difference between the LUSC and lymphoblastoid eigenvectors (requiring ≥ 0.7 for closed-in lymphoblastoid and ≤ 0.3 for open-in LUSC). The same criteria were used to identify differentially closed bins in the LUSC reference track (≥0.7 for closed-in LUSC and ≤0.3 for open-in lymphoblastoid). Concordant bins were identified by selecting regions where the LUSC and lymphoblast tracks were closed (≥ 0.65) or open (≤0.65). The deviation from the non-cancer reference profile was calculated for 20 cfDNA non-cancer samples and 7 LUSC samples with ichorCNA > 0.05 (49). The LUSC component was defined as the bin-wise median deviation of the 7 LUSC samples from the non-cancer reference. To obtain a sample-level summary of similarity to the reference tracks, we averaged the deviations for altered bins (open-in lymphoblast and closed-in LUSC, or closed-in lymphoblast and open-in LUSC) and concordant bins (open-in lymphoblast and open-in LUSC, or closed-in lymphoblast and closed-in LUSC).

### Machine Learning and Classifier Training

Using 576 donors, a machine learning model was trained to predict lung cancer status. The classifier is a Bayesian logistic regression model with prior distributions for the regression coefficients (parameters) selected to provide regularization via an L2 norm constraint to mitigate overfitting; all regression coefficients, including the intercept, were given independent Normal priors with mean = 0 and standard deviation = 1.

The joint posterior distribution of all regression coefficients given the genomic features and binary cancer status was estimated using Hamiltonian Monte Carlo, producing a set of 4,000 posterior samples of all regression coefficients from four independent chains. Each chain yielded 1,000 samples from the joint posterior distribution after discarding 1,000 “warm-up” samples.

### Statistical Analyses

The evaluable analysis population was divided using split-sample randomization in Group B (⅗ to training and ⅖ to validation) and a temporal split sample in Group A (in which the split was dictated by power requirements in the validation set, which implied a requirement of approximately 100 controls and sufficient lung cancer cases to result in approximately 100 Stage I lung cancer cases). This process afforded us six batches of distinct participants from Groups A and B to be used for held-out clinical validation, none of which were used in training (Supplementary Tables S5 and S6; Supplementary Methods). For blinding, individuals performing analyses, sequencing, and quality assessment of assay data were blinded to clinical information (aside from sex). Individuals involved in collecting and cleaning clinical information were blinded to assay data. Half of the blinded CV batches were unblinded before classifier lock and were used to confirm that cross-validated performance would generalize to an external validation set. Performance was consistent between cross-validation and these CV batches, assessed via stage-weighted sensitivity (*P* = 0.29). After this confirmation, we locked the classifier and evaluated the entire CV.

Screening population sensitivity with bootstrapped 95% confidence intervals was calculated by combining stage-specific sensitivities with the relative proportion of that stage of disease seen in the intended use population (the lung cancer screening population), as derived from the first screen year of the NLST LDCT arm (4): 54.6% Stage I, 7.5% Stage II, 21.8% Stage III, and 16.0% Stage IV. Screening population specificity with bootstrapped 95% confidence intervals was adjusted to the age distribution in the screening eligible population reported in the 2015 NHIS. Other proportion confidence intervals were calculated using the Wilson score method. Sensitivity and specificity estimation was based on complete data. Other secondary calculations were based on available data. No imputation was performed.

### Modeling Population Health Benefits of a Blood-Based Lung Screening Test

Monte Carlo simulation was conducted on a 15-mol/L person synthetic population representative of screening eligibles in the United States. Outcomes, including lung cancer screenings, lung cancer diagnosis by stage, and deaths due to lung cancer, were probabilistically generated and summed—results were then used to calculate the number of LDCT screenings required to diagnose lung cancer. The population was cycled and aged in annual increments over 5 years.

Three scenarios were considered:

(1) “Base case”: Annual LDCT screening of 6% of the population, rising to 9% in year 5 linearly (10, 50).
(2) “Base case” plus “Low” test utilization: On top of LDCT use in the base case, blood–based screening of an additional 10% of eligible patients rising to 25% in year 5 linearly. That is, net screening in year 1 is 16% (6% LDCT and 10% blood-based) and 34% in year 5 (9% LDCT screening and 25% blood-based).
(3) “Base case” plus “High” test utilization: On top of LDCT use in the base case, blood-based screening of an additional 20% rising to 50% in year 5 linearly (i.e., net screening is 26% in year 1% and 59% in year 5).

The primary analysis assumed that 80% and 10% of test positives and negatives received subsequent LDCTs, respectively. We varied this assumption in sensitivity analyses. Model inputs were derived from Surveillance, Epidemiology, and End Results (SEER) data (SEER Datasets and Software, RRID SCR_003293) and published clinical trials of LDCT (4). The simulated population was drawn from the population smoking and age distribution created by the Smoking History Generator (CISNET Publication Support and Modeling Resources, RRID: SCR_025387) to match the US population eligible for LDCT screening, specifically, those ages 50 to 80 with 20+ pack-years who either currently smoke or had quit within 15 years (51).

Individuals could age into and out of the model. Transitions to new states—a lung cancer diagnosis, death from a cause other than lung cancer, and survival with or without screening—occurred annually (Supplementary Fig. S3). Lung cancer events were assigned if a random number from 0 to 1 fell at or below that individual’s 1-year probability of developing lung cancer. This probability was derived from a validated lung cancer risk prediction model, with probabilities inflated by 1.18× to align with the increase in incidence associated with active lung cancer screening (52, 53).

The stage at diagnosis for screen-detected cancers was based on data from the NLST and outside of screening from the SEER Program, which was also the source for cancer-specific and all-cause mortality probabilities (SEER Datasets and Software, RRID SCR_003293; ref. 4). LDCT performance was based on published findings from the NLST study (4). Blood test performance was per the clinical validation findings of 80% sensitivity and 53% specificity.

Annual patient flow through the screening pathway is described in detail in Supplementary Fig. S4A and S4B. In the model, assumed screening rates are compared with randomly generated numbers to determine whether the individual underwent screening in a given year and further randomization is used to simulate the outcome of testing for the given individual.

### Bioinformatic and Statistical Software

All statistical analyses were conducted using R version 4.2. After trimming adapter sequences using fastp (0.23.2), we used Bowtie2 (2.4.2) to align paired-end reads to the hg19 reference genome. PCR duplicates were removed using samtools (1.13), and the remaining aligned read pairs were converted to a bed format using bedtools (2.27.1). Fragments were tiled into 5-Mb windows along the genome. The R packages rsample (1.0.0), recipes (1.0.1), and rstan (2.21.5) were used to implement cross-validation and to perform model training. To model the population health benefits of blood-based tests, SEER*Stat software version 8.4.0.1 was used to calculate lung cancer stage distribution for simulated nonscreen-detected cancer cases.

### Data Availability

Sequence data used for feature identification are available at the European Genome-Phenome Archive (EGA) database under the accession codes EGAS00001005340 and EGAC00001001180 as previously described (24, 26). Some patient sequence data are not publicly available due to IRB restrictions. A/B compartment chromatin data used in this study are available at https://github.com/Jfortin1/TCGA_AB_Compartments/blob/master/data/lusc_tumor_compartments_100kb.txt (LUSC) and https://github.com/Jfortin1/HiC_AB_Compartments/blob/master/data/hic_compartments_100kb_ebv_2014.txt (lymphoblastoid). Population health data were obtained from SEER data (46), data from the NLST (4), and the Smoking History Generator (CISNET Publication Support and Modeling Resources, RRID: SCR_025387). The remaining data are available within the article or Supplementary Tables.

## Supplementary Material

Supplementary Methods Additional detailed methods

Supp Figs (Fig S1-4) and Tables (Tables S1-4) Cross-validated test; DELFI score distribution; Annual transitions.

Supplementary Tables S5 and S6 Whole genome cfDNA analyses. Clinical and demographic characteristics of individuals analyzed.
