# Supplementary material for: Clinical Validation of a Cell-Free DNA Fragmentome Assay for Augmentation of Lung Cancer Early Detection
Source: Cancer Discov. 2024 Jun 6;14(11):2224–42. doi: 10.1158/2159-8290.CD-24-0519 (PMC11528203; doi:10.1158/2159-8290.CD-24-0519)
Supplement: Supplementary Methods — Additional detailed methods [file cd-24-0519_supplementary_methods_suppsm.pdf]

Supplementary Methods

Contents

I. Overview of Participant Enrollment **2**
II. DELFI-L101 Study Details **2**
A. Overview of DELFI-L101 Inclusion/Exclusion Criteria 2
B. Details of Participant Enrollment and Allocation to Participant Groups 6
C. Methods to Create Distinct Training and Validation Sets 7
III. Monte Carlo Modeling to Examine Population Health Benefits of a Blood-Based Lung Screening Test **9**
A. Methods Overview 9
B. Model Structure 9
1. Model Specifications 9
2. Patient Population 9
3. Model Flow and Transitions 11
4. Cancer Incidence 12
5. Screening Process 12
6. Screening Tests 14
7. Cancer Consequences 15
8. Model Inputs 15
9. Stage Distribution 16
10. Screening Uptake 16
11. Screening Test Characteristics 17
12. Patient Behavior Following Blood-based Test 17
13. Survival 18
IV. References **19**

I. Overview of Participant Enrollment

A. The DELFI L101 study is a multi-center, observational, case control trial with the primary objective to train and validate a lung cancer classifier.

B. The participants in the study are drawn from three separate studies that were enrolling in parallel: a DELFI sponsored protocol and two institutional protocols.

   1. DELFI sponsored protocol (NCT04825834)
   2. Institutional protocols
      a) Memorial Sloan Kettering (MSK) Lung Cancer Training Study, (NCT01775072).
      b) New York University (NYU) Lung Cancer Biomarker Center (LCBC), (NCT00301119).

C. Included in these analyses are those individuals enrolled for lung cancer training and validation which were 'cases' with lung cancer and 'controls' without lung cancer, inclusive only of those whose enrollment CT scan was conducted for lung cancer screening described in more detail in Supplemental Methods Section IIB below.

D. Not included in these analyses are participants enrolled with cancers other than lung cancer, participants without cancer whose enrollment CT was conducted to evaluate active symptoms or signs of disease (i.e. 'non-screening' participants), and those whose cancer/non-cancer status was unresolved at the time of data lock.

E. Participants enrolled through the two institutional protocols (MSK and NYU) were included in classifier training and/or validation only if they met inclusion and exclusion criteria of the L101 study and had blood samples obtained in a manner that matched the L101 sample collection protocol.

II. DELFI-L101 Study Details

A. Overview of DELFI-L101 Inclusion/Exclusion Criteria

The DNA Evaluation of Fragments for Early Interception - Lung Cancer Training Study (DELFI-L101 Study) is a multi-site, prospectively enrolling, observational, case-control study. Participants were enrolled from a variety of clinical sites, including lung cancer screening clinics, pulmonary/lung nodule clinics, thoracic

surgery clinic, or cancer centers from both community and academic health systems.

Eligibility criteria were developed to mimic the intended use population for a lung cancer screening test and therefore be representative of the elevated risk population eligible for lung cancer screening based on the 2021 United States Preventive Services Task Force (USPSTF) criteria. Specifically, inclusion criteria were age $\geq$ 50 years and smoking history of $\geq$20 pack-years. There was no upper age limit or limit on the number of years a participant had quit smoking. CT of the thorax within 12 months of enrollment or planned within 6 weeks was also required.

For the development and validation of the lung classifier, participants were eligible if they had:

- no suspected or confirmed lung cancer diagnosis as defined by no clinical and/or radiological findings that indicate suspicion of lung cancer diagnosis or
- suspected of lung cancer as defined by radiological finding and/or clinical evaluation that indicates suspicion of lung cancer diagnosis or
- confirmed, untreated lung cancer as defined by pathologic diagnosis of lung cancer with no prior systemic therapy, definitive therapy, radiation, or surgical resection for any lesion.

Exclusion criteria included: prior cancer therapy within one year of enrollment for any cancer other than biopsies or treatment of non-melanotic skin cancer, any history of hematologic malignancies or myelodysplasia, or organ transplantation, history of blood product transfusion within 120 days prior to enrollment, current pregnancy.

The participants in the DELFI L101 study were drawn from three separate studies that were enrolling in parallel: a DELFI sponsored protocol (NCT04825834) and two institutional protocols.

Memorial Sloan Kettering (MSK) Lung Cancer Training Study (NCT01775072)

was approved by the MSK IRB (Protocol #22-273) is an umbrella protocol that allows enrollment of participants with known cancer, undergoing work-up to confirm or exclude a cancer diagnosis, and those at increased risk due to family history, genetic, or lifestyle factors.

NYU Lung Cancer Biomarker Center (LCBC) protocol (NCT00301119) approved by the NYU IRB (Protocol #8896) is a study designed to investigate biomarkers in screening participants for lung cancer. Two groups of patients are included: (1) a screening cohort that undergoes questionnaires, pulmonary function testing, CT scan, sputum induction and phlebotomy, and (2) a rule-out lung cancer group that undergoes the same procedures, but in addition, has a diagnostic work-up with bronchoscopy and/or surgical lung resection.

The DELFI-L101 inclusion and exclusion criteria were applied consistently across all enrolling protocols to confirm eligibility for enrollment in the study. A detailed DELFI-L101 study enrollment flow diagram is shown in **Supplementary Methods Figure 1**.

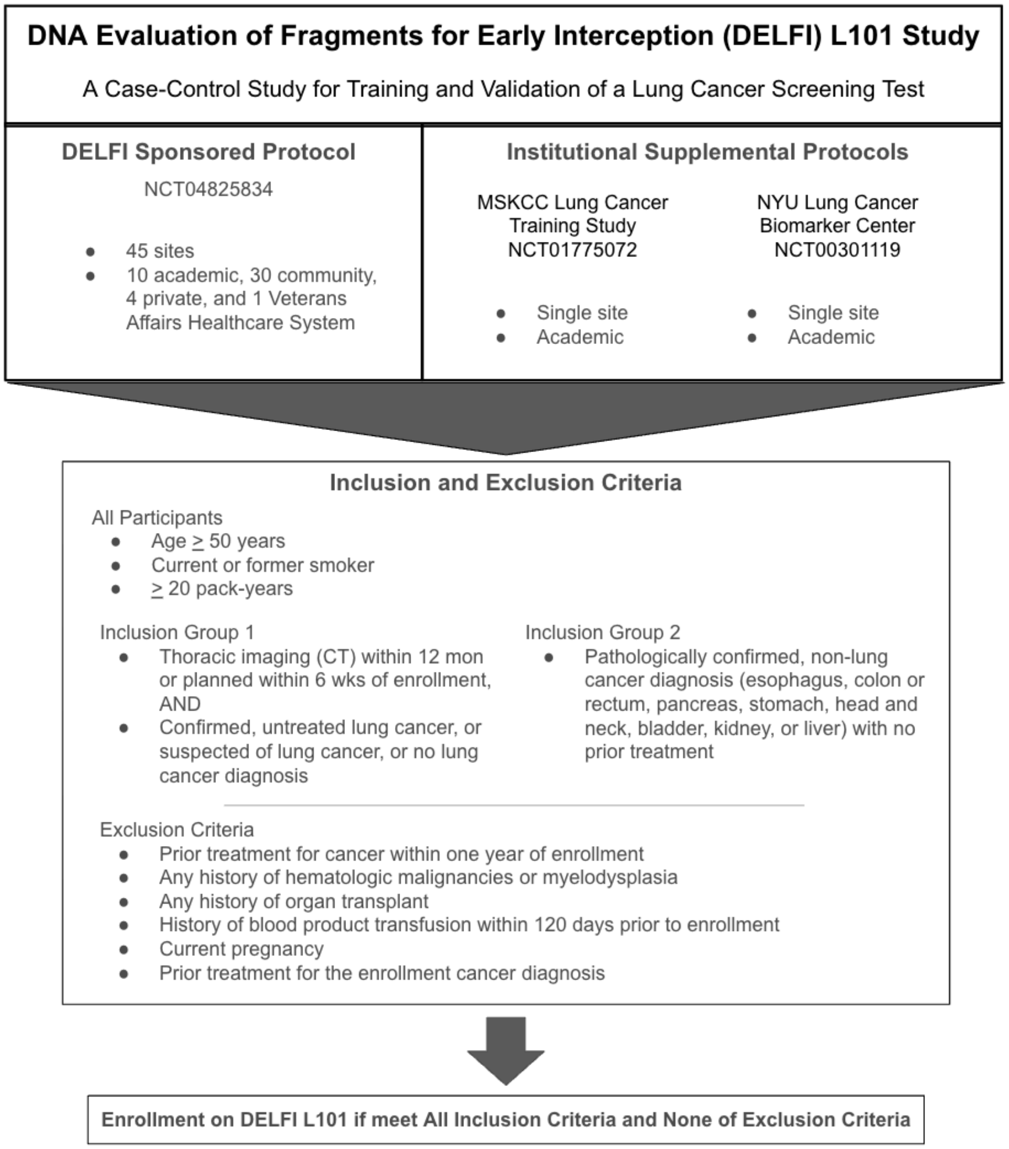

Supplementary Methods Figure 1. The DNA Evaluation of Fragments for Early Interception (DELFI) L101 study (NCT 04825834) is an observational, case-control study that prospectively enrolled participants from 47 sites across the US. The purpose of the study is to train and validate a classifier for lung cancer detection using the DELFI assay in the development of a blood-based lung cancer screening test. Per the master DELFI-L101 sponsored protocol (NCT04825834), all inclusion and none of the exclusion criteria were required to be met by each participant for enrollment onto the study, including those participants enrolling under supplemental institutional protocols (NCT01775072 and NCT00301119).

B. Details of Participant Enrollment and Allocation to Participant Groups

All participants enrolled in the DELFI-L101 study were assigned a Group label. Group A consisted of those participants with pathologically confirmed lung cancer at enrollment. Group B consisted of participants without a cancer diagnosis and without any suspicion for lung cancer at enrollment. Group Indeterminate consisted of participants who were suspected of lung cancer at enrollment, defined as any radiological finding and/or clinical evaluation that indicates suspicion of lung cancer diagnosis (i.e., suspicious lung nodule(s) ≥6mm or Lung-RADS 3/4 for first-time CT scan patients and newly suspicious lung nodule(s) ≥6mm or Lung-RADS 3/4 identified within 6 months prior to enrollment for follow-up and surveillance CT scan patients). Group C consisted of participants with confirmed non-lung cancers. For the purposes of developing and validating a lung cancer classifier, only participants enrolled in Group A and Group B were considered for inclusion in the analysis as shown in **Supplementary Methods Figure 2**.

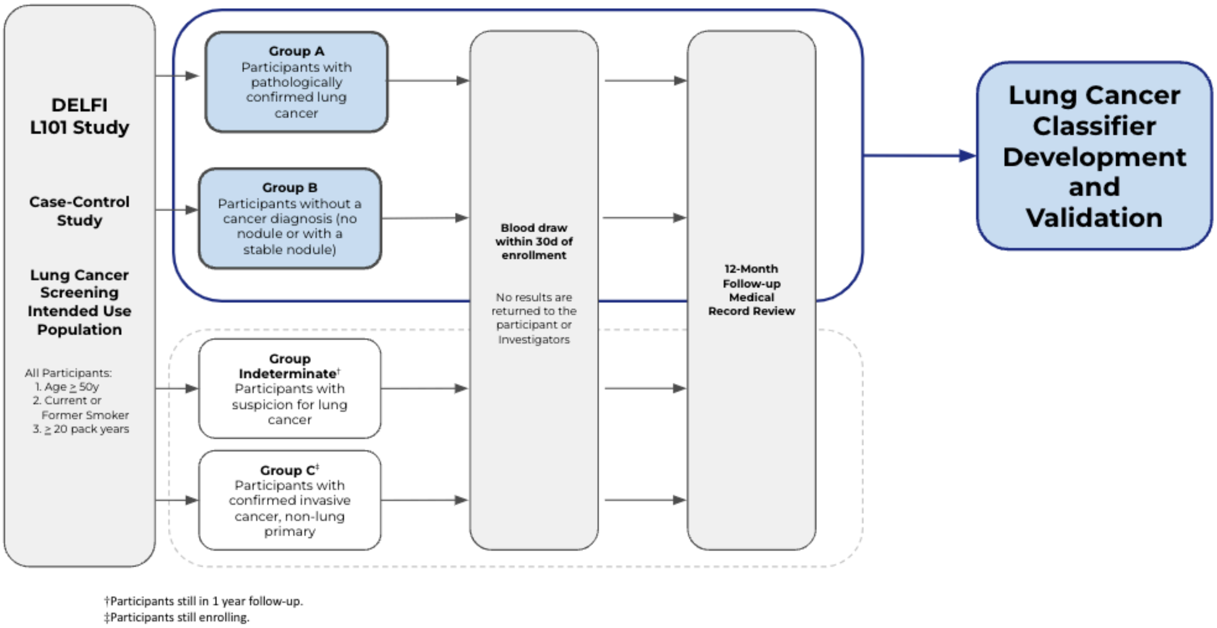

Supplementary Methods Figure 2. The DELFI L101 case-control study enrolled participants into one of four groups: (1) Group A, lung cancer cases, (2) Group B, participants without cancer, (3) Group Indeterminate, participants with a suspicion of lung cancer, and (4) Group C, non-lung

cancer cases. For the purposes of the development and validation of a lung cancer classifier, only participants from Group A and Group B were considered for inclusion in the analysis.

To be considered evaluable for the purpose of classifier training and validation, additional exclusion criteria were applied. Participants were not considered evaluable if after enrollment they failed inclusion/exclusion criteria, were unable to have a group assigned, had a protocol deviation, or were part of a non-screening population. The non-screening population exclusion criteria was applied to ensure that the participant's enrollment and blood sample collection occurred under the conditions and processes that were anticipated for future screening implementation and use of the test. As a result, participants without cancer (Group B) whose enrollment CT was conducted to evaluate active symptoms or signs of disease were excluded (i.e. 'non-screening' participants). Additionally, participants that had blood samples obtained in a manner that did not match the sample collection protocol for peripheral venipuncture (eg. central line collection) were excluded (i.e. 'non-screening' participants).

C. Methods to Create Distinct Training and Validation Sets

1. Sample size requirements for clinical validation: The relative allocation of Group A and Group B participants to training and validation sets were determined based on the sample size requirements for estimating test specificity and sensitivity (both overall and for Stage I lung cancer) in clinical validation. These statistical needs led to fewer overall Group B than Group A members being allocated to the validation set, while the reverse was true for allocation to the Classifier Training set.
2. Participants in the validation set were selected based on a temporal split in the collection of samples in both the cancer and non-cancer cohorts. DNA extraction, library preparation, and sequencing for 576 samples in the training cohort were performed prior to August 2022, whereas the 382 participants in the validation cohort were not processed until after April 2023 (Supplementary Table 1).
3. All told, there were a total of 18 distinct batches of participants, including 12 batches for training, and 6 batches for validation that were stratified on

Lung Cancer status and stage. The validation set batches were not used in any manner to train the classifier, and the results reported here are those for the locked classifier validated on these batches. This also generated a validation set that could be partially unblinded to confirm the generalizability of the trained classifier before classifier lock without allowing any information from the validation set to be incorporated into the training of the classifier.

4. The sensitivity and specificity of the classifier in the clinical validation set characterizes the test performance on a future collection of samples from the intended use population, as required.

III. Monte Carlo Modeling to Examine Population Health Benefits of a Blood-Based Lung Screening Test

A. Methods Overview

In this study, we developed a Monte Carlo Simulation model to assess population health outcomes associated with incorporating the blood-based test into the screening pathway for those who are eligible but not undergoing annual LDCT screening. A Microsoft Excel-based, interactive model was developed to consider USPSTF eligible individuals and simulate them for a five-year period. Clinical outcomes included the number of cancers detected, the proportion of detected cancers diagnosed at each clinical stage, and the number of cancer deaths.

B. Model Structure

1. Model Specifications

The model developed for this analysis was a Monte Carlo Simulation model programmed in Excel, utilizing Visual Basic for Applications (VBA) to assess eligible patients. As a Monte Carlo model, individuals were simulated through the model one-at-a-time, with outcomes determined for each individual and summed across the cohort through random number generation and probability cutoffs. The model cycled the model population through five one-year cycles to estimate incidence, and used findings to project outcomes.

2. Patient Population

We considered a US population with no history of lung cancer eligible for LDCT screening, specifically those ages 50-80 with 20+ pack years who either currently smoke or had quit within the past 15 years. The cohort was derived from the synthetic population generated by the Smoking History Generator (SHG)[1]. The SHG is a tool developed as part of the Cancer Intervention and Surveillance Network (CISNET) and combines data from multiple sources to create individuals often used for simulations to assess the impact of different approaches to lung cancer screening[1,2].

Data generated by the SHG include age, sex, average pack years, and quit age. We obtained files for each birth cohort, by gender, from 1942-1982, and then sampled those eligible to create our model population. The process for synthesizing data provided by the CISNET group is described in **Supplementary Methods Table 1**.

**Supplementary Methods Table 1. Analysis of Smoking History Generator**
To create a single file with individuals representative of the current USPSTF-eligible population the following analytic plan was performed.
Analytic Plan:<br>Drop **Race** (column 1)<br>Generate **Age as of 2022** = 2022 – Birth cohort (column 3)<br>Keep **Sex**<br>Generate **Cigarettes per day** = Average of columns 8,10,12, etc. if:<br>the column value is larger than 0, AND<br>the previous column is less than or equal Age as of 2022<br>Generate **Pack year** = Sum columns 8,10,12, etc. if:<br>the column value is larger than 0, AND<br>the previous column is less than or equal Age as of 2022<br>then divide the sum by 20<br>Generate **Duration of smoking** = Minimum of<br>Age as of 2022 – age at starting smoking, AND<br>Age at quitting smoking – age at starting smoking<br>Generate **Years since quit** = Minimum of<br>Zero, AND<br>Age as of 2022 – age at quitting smoking<br>Generate **Calendar year at quitting** = Birth year + age at quitting<br>Generate incremental smoking years future model years.<br>4 variables for 2023-2026 indicating additional smoking years from model start<br>If age at quitting smoking ≤ age as of 2022, then zero<br>Otherwise, in year y (y in 2023 – 2026) the value is the minimum of<br>Year at quitting – 2022, AND<br>Y – 2022<br>Drop variables in column 7 and on in the original files<br><br>Exclusion criteria<br>Age:<br>Drop individual if Age as of 2022 < 45<br>Drop individual if Age as of 2022 > "Other cause mortality age"<br>Minimum pack year<br>Drop individual if Pack year < 20

An open cohort approach was taken in which individuals could age-in to the model (i.e., become eligible for screening after the first year), such

that someone age 48 years at the beginning of the model would begin being assessed in the third year when they turned 50 years. Similarly, patients aged-out of the model (i.e., stopped being considered in calculations) after age 80.

3. Model Flow and Transitions

   The model starting population was US adults without diagnosed cancer who were recommended to undergo LDCT. Each year, individuals without detected cancer could be diagnosed with cancer, die from non-cancer causes, or survive (with or without having undergone screening) and continue to the next year to repeat the process. Those who were diagnosed with cancer were removed from the annual screening process and could survive with cancer or die from either cancer or non-cancer causes. A simple diagram showing this process is found in **Supplementary Figure S3**.

4. Cancer Incidence

   To determine whether an individual developed cancer each year, we utilized the Bach risk equation, a previously developed risk prediction model[3]. To apply the equation we assumed 1.5% of eligible individuals had been exposed to asbestos based on a prior publication[4]. To account for the fact that LDCT screening increases the likelihood of diagnosis, we applied a 1.18x incidence inflation factor based on prior literature[5]. This calculated risk of cancer for the individual was then compared to one or more random numbers during the screening process to determine whether the underlying cancer was detected via screening, detected via non-screening methods, or remained undetected.

5. Screening Process

   Each year, individuals in the appropriate age range without detected cancer would be eligible for screening. Prior to consideration of whether screening would occur, a random number would be generated to

determine whether they had a tumor that could potentially be detected. Independent of underlying tumor status, a random number was then generated to determine whether they would undergo screening (either with LDCT or a blood-based test). For those not undergoing screening and without an underlying cancer, they would face a risk of dying from other causes. Those who survived would then repeat this process the following year. For those not undergoing screening and having an underlying cancer, there would be a probability that the cancer was not detected that year and that individual would have a risk of death from other causes and otherwise continue to the next year. For those undergoing screening, diagnosis of cancer would only come following a positive LDCT amongst those with cancer which could take place as the only screening test or be conducted following the blood-based test. Both the blood-based test and LDCT were assumed to be imperfect in terms of sensitivity and specificity. Those who had screened negative but had an underlying cancer (i.e., false negatives) were still eligible to be diagnosed with lung cancer in the same year. A schematic of the screening process is shown in **Supplemental Methods Figure 3**.

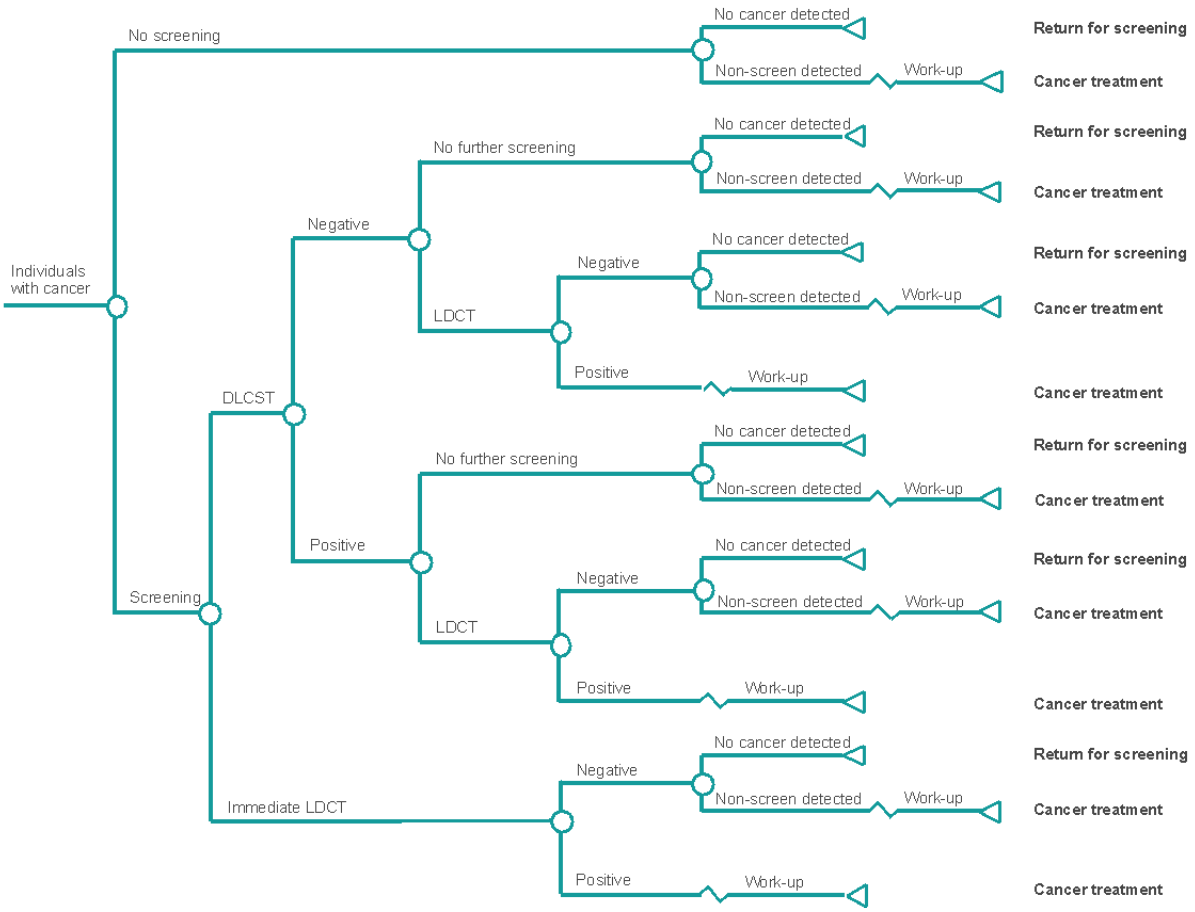

Supplementary Methods Figure 3. Screening Process for Individuals with Cancer. Flow diagram demonstrating the decision branches during the screening process for individuals within the simulated population who have cancer. Symbols: Circle: chance or decision node; Triangle: end nodes; Solid line: standard decision tree branch.

6. Screening Tests

The model considered both LDCT and the blood-based test, and the share of the population receiving each was explored. In the scenario without the blood-based test, LDCT was the only option and everyone who was screened underwent that test. In the scenario with the blood-based test, individuals could either go directly to LDCT or could first get a blood-based test. Following either a negative or positive blood-based test, patients could either next undergo an LDCT, or could

end the screening process for the year.

7. Cancer Consequences

   Cancer patients had risk of mortality applied, both cancer-specific and other-cause mortality which were determined from the literature on stage-specific survival rates[6].

8. Model Inputs

   To project the impact of screening on cancer incidence and mortality, the model required parameters related to the following:

   - The distribution of patients across cancer stages at detection
   - Disease- and test-characteristics
   - Clinical outcomes

   All model-required inputs (shown in **Supplemental Methods Table 2**) were derived from the literature, analyses of primary data from the National Cancer Institute's SEER database, the National Lung Screening Trial and other sources[6,7].

Supplemental Methods Table 2. Model Inputs

Model Input Categories
Eligible Patient Population Age, Smoking Status, Smoking Intensity
Stage Distribution at Detection
Rate of Overdiagnosis
LDCT and DLSCT Test Characteristics and Uptake
Patient Behavior Following a Blood-based Test
Survival

9. Stage Distribution

   The proportion diagnosed at each stage of lung cancer for screen detected cancers was based on data from the LDCT arm of the National Lung Screening Trial (NLST), incorporating the impact of screening

‘round’ (whether it was the patient’s first, second, or third screen)[7]. Stage distribution for non screen detected lung cancers were derived from the Surveillance, Epidemiology, and End Results (SEER) Program. While this has a slight limitation in that the model is using these estimates to inform the non-screen detected distribution while a small percentage of the registry data is from patients who were screen detected, most cancer cases detected currently are not via LDCT and the large sample and generalizability of SEER outweighed this limitation. Inputted stage distributions for each method of detection are shown in **Supplemental Methods Table 3**.

Supplemental Methods Table 3: Cancer Stage at Diagnosis by Method of Detection

	Stage 1	Stage 2	Stage 3	Stage 4
Patients Screen Detected	63.0%	7.2%	17.0%	12.8%
Patients Non-Screen Detected	22.8%	4.7%	24.6%	47.9%

10. Screening Uptake

The model assesses three scenarios. A ‘base case’ where only LDCT is utilized and its utilization matches current rates in the US with increases in each year (from 6% to 9% linearly). The second two scenarios incorporate the blood-based test at relatively low use rising slowly (the ‘low’ scenario from 16% to 34% linearly) and a somewhat more robust utilization rising more quickly (the ‘high’ scenario from 26% to 59% linearly). The model assumes at the individual level that screening adherence is independent each year.

11. Screening Test Characteristics

The sensitivity and specificity of both LDCT and the blood-based test were incorporated to capture the proportion of individuals who would be correctly identified as having or not having lung cancer. Such test

characteristics for LDCT were based on an analysis of NLST data[8], and from the clinical validation data for the blood-based test. Test characteristics are found in **Supplemental Methods Table 4**. It is assumed that the diagnostic workup process following a positive LDCT can perfectly differentiate those with and without cancer and is done so within a one-year period.

Supplemental Methods Table 4: Screening Test Characteristics

	Blood-based Test	LDCT
True Positive Rate	0.80	0.93
False Positive Rate	0.42	0.24
Positive Predictive Value (PPV)	1.2%	2.2%
Negative Predictive Value (NPV)	99.8%	99.9%

12. Patient Behavior Following Blood-based Test

To capture expected real-world utilization of screening tests, we estimated 80% of those with a positive result from the blood-based test and 20% of those with a negative result from the blood-based test would go on to receive a LDCT.

13. Survival

To estimate the change in lung cancer deaths under different scenarios, we incorporated mortality rates for those without cancer, as well as both cause-specific and all-cause mortality rates for those with cancer (shown in **Supplemental Methods Table 5**). All-cause mortality rates for those without cancer during the five-year model period were based on projections from the SHG[1]. For those with cancer, cancer-specific and all-cause mortality during the five-year model period was based on data from SEER[6]. To match observed data from NLST, based on empiric comparisons, screen vs. non-screen detected lung cancers were set to have similar outcomes for stages II-IV. For stage I cancers, the mortality for those non-screen detected were based on SEER, while the screen detected mortality rates for Stage I patients from SEER were reduced to account for those over-diagnosed, based on observed data from NLST[9].

Supplemental Methods Table 5: Annual Mortality Probability Within Model Time Horizon

Stage	Mortality Type	Year 1	Year 2	Year 3	Year 4	Year 5
**1**	**Cancer Specific: Screen Detected**	5.8%	5.4%	4.4%	3.5%	2.9%
**1**	**All-Cause: Screen Detected**	10.2%	8.8%	8.1%	7.2%	7.1%
**1**	**Cancer Specific: Non-Screen Detected**	10.1%	9.5%	7.6%	6.2%	5.1%
**1**	**All-Cause: Non-Screen Detected**	14.5%	12.9%	11.4%	9.9%	9.3%
**2**	**Cancer Specific**	21.8%	20.4%	15.8%	11.2%	8.8%
**2**	**All-Cause**	26.1%	23.4%	19.0%	14.5%	12.0%
**3**	**Cancer Specific**	44.0%	35.1%	24.0%	16.9%	13.2%
**3**	**All-Cause**	47.6%	37.7%	27.0%	20.4%	17.0%
**4**	**Cancer Specific**	71.8%	51.8%	35.4%	26.0%	19.9%
**4**	**All-Cause**	73.7%	53.6%	37.8%	28.5%	23.0%

IV. References

1. CISNET Publication Support and Modeling Resources. *US Department of Health and Human Services*.
2. Krist, A. H. *et al.* Screening for Lung Cancer: US Preventive Services Task Force Recommendation Statement. *Jama* **325**, 962–970 (2021).
3. Bach, P. B. *et al.* Variations in Lung Cancer Risk Among Smokers. *Jnci J National Cancer Inst* **95**, 470–478 (2003).
4. Araki, T. *et al.* Pleural abnormalities in the Framingham Heart Study: prevalence and CT image features. *Occup. Environ. Med.* **74**, 756 (2017).
5. Patz, E. F. *et al.* Overdiagnosis in Low-Dose Computed Tomography Screening for Lung Cancer. *JAMA Intern. Med.* **174**, 269–274 (2014).
6. National Cancer Institute Surveillance, Epidemiology, and End Results Program. *SEER Stat Software*.
7. Aberle, D. R. *et al.* Reduced Lung-Cancer Mortality with Low-Dose Computed Tomographic Screening. *New Engl J Medicine* **365**, 395–409 (2011).
8. Pinsky, P. F., Gierada, D. S., Nath, H., Kazerooni, E. A. & Amorosa, J. ROC Curves for Low-Dose CT in the National Lung Screening Trial. *J Med Screen* **20**, 165–168 (2013).
9. Gierada, D. S. & Pinsky, P. F. Survival Following Detection of Stage I Lung Cancer by Screening in the National Lung Screening Trial. *Chest* **159**, 862–869 (2021).
