# Supplementary material for: Clinical Validation of a Cell-Free DNA Fragmentome Assay for Augmentation of Lung Cancer Early Detection
Source: Cancer Discov. 2024 Jun 6;14(11):2224–42. doi: 10.1158/2159-8290.CD-24-0519 (PMC11528203; doi:10.1158/2159-8290.CD-24-0519)
Supplement: Supp Figs (Fig S1-4) and Tables (Tables S1-4) — Cross-validated test; DELFI score distribution; Annual transitions. [file cd-24-0519_supp_figs_fig_s1-4_and_tables_tables_s1-4_suppsf1-sf4st1-st4.pdf]

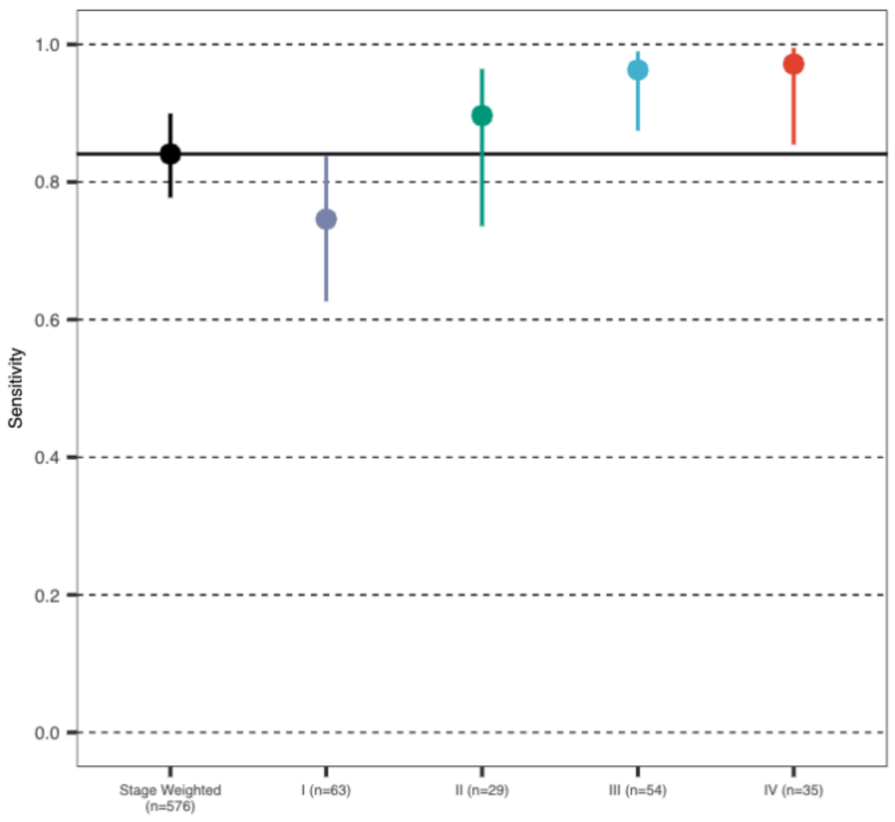

Supplementary Figure S1 Cross-validated test performance in the training set. Sensitivity of the test in the classifier training set (N =576) by cancer stage and overall. Point estimates are reported with 95% Wilson confidence intervals.

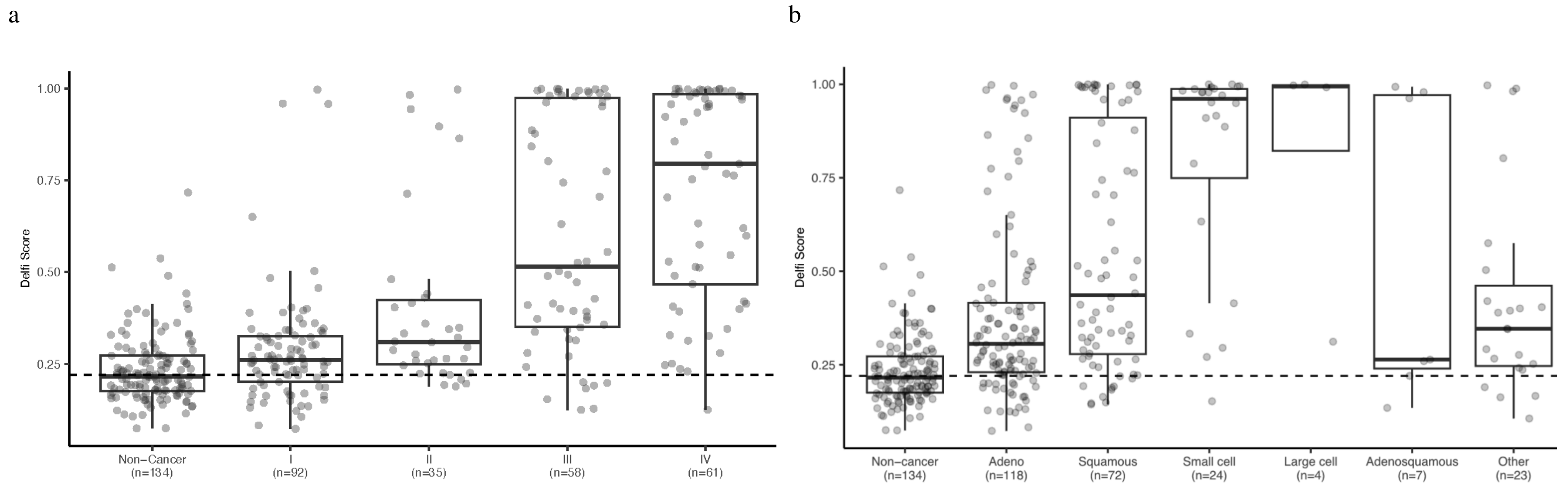

Supplementary Figure S2 DELFI score distribution in the L101 clinical validation set. a, Boxplots indicate the interquartile range and median value (horizontal line) of DELFI scores for individuals without cancer and for individuals with cancer stratified by stage (n=380, 2 participants excluded due to unknown stage). The boxplots suggest a gradual and then sharp increase in the DELFI score distribution as cancer progresses from early to late-stage disease. **b,** Score distributions stratified by histology indicate that small cell carcinomas have the highest separation from non-cancer scores, while adenocarcinomas and squamous cell carcinomas tend to have lower scores.

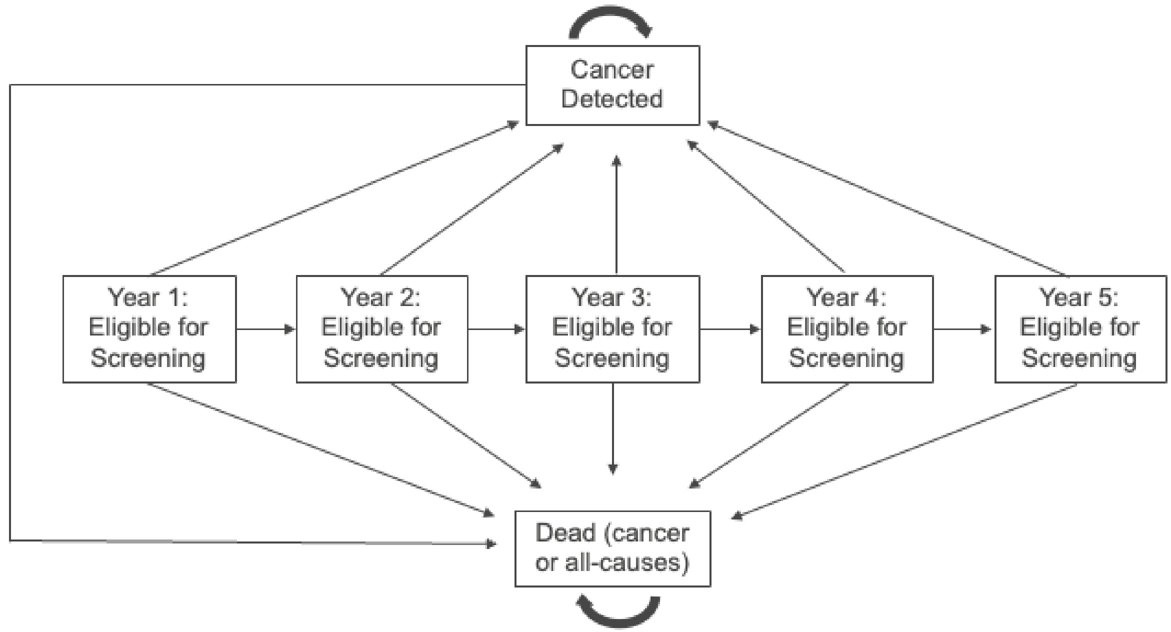

Supplementary Figure S3 Annual transitions for population health modeling scenarios. Patients enter the model in Year 1 eligible for screening (no detected cancer). Each year they can be detected with cancer, die, or have no cancer detected and return for screening the following year. Those with cancer detected are no longer screened and remain in the Cancer Detected state until death. Those below the eligibility age in year 1, but reaching that age during the 5 years, will begin screening once appropriate.

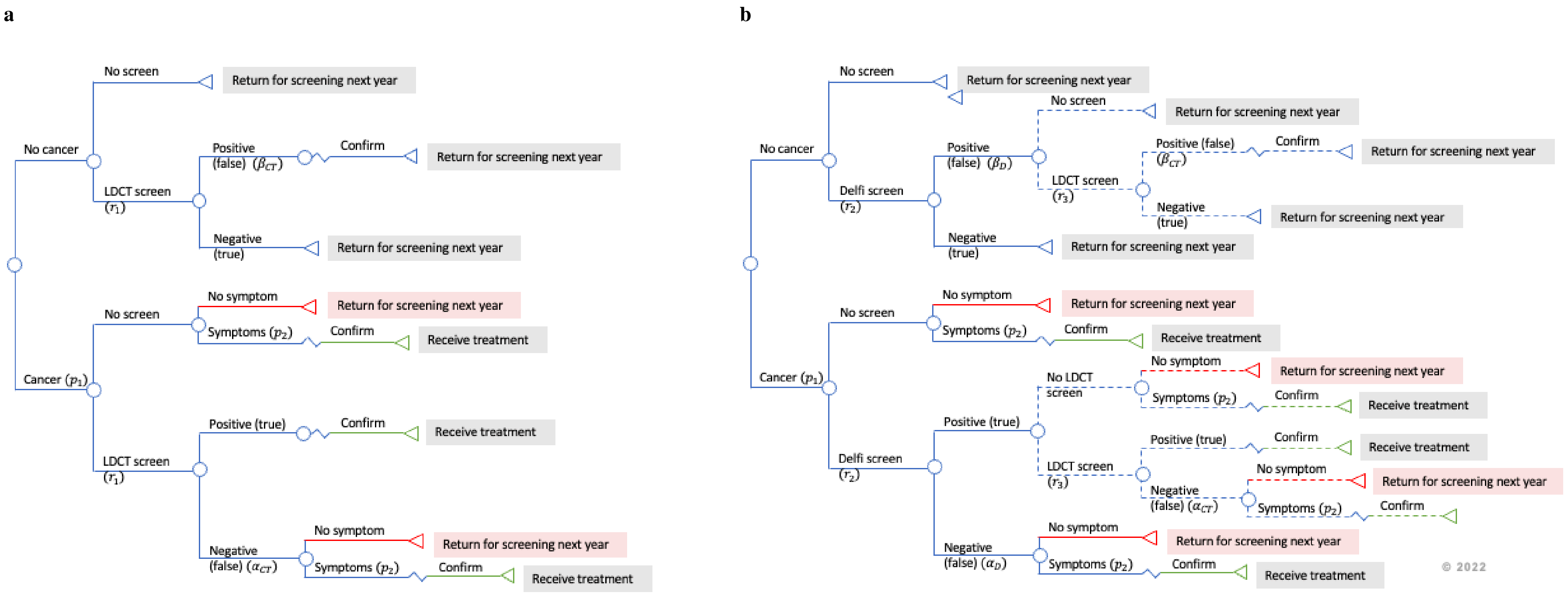

Supplementary Figure S4 Within-year flow for population health modeling scenarios. **a,** Flow diagram demonstrating the potential within-year patient dispositions for LDCT alone scenario. **b,** Flow diagram demonstrating the potential within-year patient dispositions for blood test scenarios. Symbols: Blue line: standard decision tree branch; Red line: decision tree branches leading to false negative diagnosis; Green line - decision tree branches leading to true positive diagnosis; Circle: chance or decision node; Triangle: end nodes; Solid line: standard decision tree branch; Dashed line: additional decision tree with introduction of blood test; p_1: probability of cancer; r_1: rate of patients undergoing LDCT screen if blood test is not available; r_2 – rate of patients undergoing blood test; r_3 – rate of patients undergoing LDCT if blood test comes back positive; p_2 – probability of symptoms in given year given the patient has cancer; α_{CT} – rate of false negative (negative test result if the patient has cancer) for LDCT; β_{CT} – rate of false positive (positive test result if the patient has no cancer) for LDCT; α_D - rate of false negative for blood test; β_D - rate of false positive for blood test.

Supplementary Table S1. Participant Demographics and Clinical Characteristics by L101 Case and Control Status

**Supplementary Table S1. Participant demographics and clinical characteristics**					
	**L101 Study**		**NHIS 2015***	**NLST, Both Arms****	**Silvestri et al.*****
**Characteristics**, Median (IQR); n (%)	Cases (n= 429)	Controls (n= 529)	(n= 2,261) (Weighted N= 13,975,210)	(N=53,452)	(N=1,052,591) [USPSTF 2013]
**Age** (years), median (Q1, Q3)	70 (64, 75)	64 (59, 70)	60 (55, 67)	60.0 (57.0, 65.0)	-
**Age**, N (%)					
Age < 65 years	116 (27%)	267 (50%)	9,318,186 (67%)	39,234 (73%)	504,794 (48%)
Age >= 65 years	313 (73%)	262 (50%)	4,657,024 (33%)	14,218 (27%)	447,797 (42%)
Unknown or not reported	0 (0%)	0 (0%)	0 (0%)	0 (0%)	100,000 (10%)
**Sex**, N (%)					
Male	213 (50%)	282 (53%)	7,788,381 (56%)	31,530 (59%)	544,482 (52%)
Female	216 (50%)	247 (47%)	6,186,829 (44%)	21,922 (41%)	505,318 (48%)
Unknown or not reported	0 (0%)	0 (0%)	0 (0%)	0 (0%)	2,791 (<1%)
**Race**, N (%)					
White	375 (87%)	435 (82%)	12,313,537 (88%)	48,549 (91%)	461,593 (44%, 92% †)
Black or African American	39 (9%)	51 (10%)	1,065,711 (8%)	2,376 (4%)	37,111 (3%, 7% †)
Other Groups****	15 (4%)	43 (8%)	595,962 (4%)	2,527 (5%)	553,887 (53%, 1% †)
**Ethnicity**, N (%)					
Not Hispanic or Latino	375 (87%)	359 (68%)	13,392,848 (96%)	52,118 (98%)	400,737 (38%, 98% †)
Hispanic or Latino	17 (4%)	140 (26%)	582,362 (4%)	935 (2%)	8,807 (1%, 2% †)
Unknown or not reported	37 (9%)	30 (6%)	0 (0%)	399 (1%)	643,047 (61%, 0% †)
**Education**, N (%)					
Less than High School Graduate	15 (4%, 11% †)	43 (8%, 12% †)	851,379 (6%)	3,249 (6%)	10,743 (1%, 13% †)
High School Graduate	45 (10%, 32% †)	121 (23%, 34% †)	1,279,954 (9%)	12,712 (24%)	34,150 (3%, 41% †)
Associate Degree or Some College or Some Training after High School	43 (10%, 30% †)	99 (19%, 28% †)	5,364,443 (38%)	19,711 (37%)	24,758 (2%, 30% †)
College Graduate	22 (5%, 15% †)	68 (13%, 19% †)	4,425,167 (32%)	8,946 (17%)	13,613 (1%, 16% †)
Postgraduate or Professional Degree	17 (4%, 12% †)	25 (5%, 7% †)	2,027,572 (15%)	7,600 (14%)	
Unknown or not reported	287 (67%, 0% †)	173 (33%, 0% †)	26,695 (<1%)	1,234 (2%)	969,327 (92%, 0% †)
**Geographic Region**, N (%)					
Northeast	113 (26%)	145 (27%)	2,429,248 (17%)	8,713 (16%)	264,819 (25%)
Midwest	166 (39%)	63 (12%)	3,665,934 (26%)	20,953 (39%)	315,702 (30%)
South	96 (22%)	221 (42%)	5,517,263 (39%)	12,775 (24%)	363,509 (35%)
West	54 (13%)	100 (19%)	2,362,765 (17%)	11,011 (21%)	107,502 (10%)
**BMI**, median (Q1, Q3)	27 (23, 31)	28 (25, 33)	27 (24, 31)	27 (24, 31)	-
**Lung Cancer USPSTF 2021 Criteria**, N (%)					

USPSTF (2021)	307 (71%)	491 (93%)	13,975,210 (100%)	52,834 (99%)	1,052,591 (100%)
Non-USPSTF (2021)	97 (23%)	37 (7%)	0 (0%)	618 (1%)	0 (0%)
Unknown or not reported	25 (6%)	1 (<0.2%)	0 (0%)	0 (0%)	0 (0%)
**Pack-Years**, median (Q1, Q3)	45 (30, 64)	40 (30, 52)	38 (28, 50)	48 (39, 66)	-
**Smoking Cigarettes per Day**, median (Q1, Q3)	20 (20, 30)	20 (20, 20)	20 (15, 20)	25 (20, 35)	-
**Years Since Smoking Cessation**, median (Q1, Q3)	9 (1, 20)	7 (2, 12)	6.0 (3.0, 10.0)	7.0 (3.0, 11.0)	-
**Smoking Status, N (%)**					
Current	152 (35%)	258 (49%)	8,178,059 (59%)	25,760 (48%)	645,875 (61%)
Former	277 (65%)	271 (51%)	5,797,151 (41%)	27,692 (52%)	406,700 (39%)
**COPD Status, N (%)**					
COPD	208 (48%)	239 (45%)	2,600,068 (19%)	2,690 (5%)	-
No COPD	210 (49%)	279 (53%)	11,365,635 (81%)	50,426 (95%)	-
Unknown or not reported	11 (3%)	11 (2%)	9,507 (<0.1%)	336 (<1%)	-
**Type II Diabetes, N (%)**					
Yes	107 (25%)	110 (21%)	2,689,713 (19%)	5,174 (9.7%)	-
No	312 (73%)	411 (78%)	10,929,523 (78%)	48,047 (90%)	-
Unknown or not reported	10 (2%)	8 (2%)	355,974 (3%)	231 (<1%)	-
**Prior History of Cancer**					
Yes	101 (24%)	60 (11%)	2,184,948 (16%)	2,289 (4%)	-
No	328 (76%)	469 (89%)	11,762,865 (84%)	50,951 (96%)	-
Unknown or not reported	0 (0%)	0 (0%)	27397 (<1%)	212 (<1%)	-

Footnotes:
† Report percent (%) among those with a reported value;
*Subset to LCS-eligible survey respondents as per 2021 USPSTF guidelines, including individuals who had quit smoking; those with a history of lung cancer (unweighted n=45) were excluded.
**NLST demographics, except Geographic region, were calculated internally using NLST data. The numbers are comparable to published estimates (https://www.ncbi.nlm.nih.gov/pmc/articles/PMC2994863/).
***Silvestri GA, Goldman L, Tanner NT, et al. Outcomes From More Than 1 Million People Screened for Lung Cancer With Low-Dose CT Imaging. CHEST. 2023;164(1):241-251. doi:10.1016/j.chest.2023.02.003
****Data was aggregated when cell counts are < 11 to avoid risk of re-identification. Other Groups includes Asian, American Indian, Alaskan Native, Native Hawaiian, Other Pacific Islander, Other, Unknown, or Not Reported
USPSTF 2013 criteria for lung cancer screening: Age 55-80 years, pack-years >=30 years, and quit-years <= 15 years.
USPSTF 2021 criteria for lung cancer screening: Age 50-80 years, pack-years >=20 years, and quit-years <= 15 years.

Supplementary Table S2. Tumor Characteristics of Cases in L101 Training and Clinical Validation

**Supplementary Table S2. Tumor Characteristics of Cases in L101 Training and Clinical Validation**				
	**L101 Study**		**NLST LDCT Arm***	**Silvestri et al.**
	Cases in Training (n=181)	Cases in Validation (n=248)	T0-T2 only (N=710)	(n=5,876)
**Stage**				
I	63 (35%)	92 (37%)	406 (57%)	2,326 (40%, 53% †)
II	29 (16%)	35 (14%)	53 (7%)	551 (9%, 13% †)
III	54 (30%)	58 (23%)	132 (19%)	850 (14%, 20% †)
IV	35 (19%)	61 (25%)	105 (15%)	620 (11%, 14% †)
Unknown or not reported	0 (0%)	2 (1%)	14 (2%)	1,529 (26%, 0% †)
**Stage, IA or IB-IV**				
IA	54 (30%)	75 (30%)	329 (46%)	-
IB-IV	127 (70%)	171 (69%)	367 (52%)	-
Unknown or not reported	0 (0%)	2 (1%)	14 (2%)	
**Tumor stage**				
T1	69 (38%)	105 (42%)	448 (63%)	-
T2	34 (19%)	60 (24%)	98 (14%)	-
T3	31 (17%)	28 (11%)	10 (1%)	-
T4	21 (12%)	20 (8%)	95 (13%)	-
TX, T0, Unknown, or Not Reported	26 (14%)	35 (14%)	59 (8%)	-
**Node Stage**				
N0	86 (48%)	122 (49%)	441 (62%)	-
N1	14 (8%)	22 (9%)	30 (4%)	-
N2	41 (23%)	50 (20%)	120 (17%)	-
N3	13 (7%)	16 (6%)	41 (6%)	-
NX, Unknown, Not Reported	27 (15%)	38 (15%)	78 (11%)	-
**Metastasis Stage**				
M0	105 (58%)	131 (53%)	527 (74%)	-
M1	27 (15%)	43 (17%)	97 (14%)	-
MX, Unknown, Not Reported	49 (27%)	74 (30%)	86 (12%)	-
**Histology**				
Small Cell Carcinoma	19 (10%)	25 (10%)	66 (9%)	-
Non-Small Cell Carcinoma	162 (90%)	223 (90%)	636 (90%)	-
Unknown or Not reported	0 (0%)	0 (0%)	3 (<1%)	-
Missing	0 (0%)	0 (0%)	5 (<1%)	-
**Histology sub-type**				
Adenocarcinoma	89 (49%)	118 (48%)	354 (50%)	-

Adenosquamous Carcinoma	2 (1%)	7 (3%)	12 (2%)	-
Large Cell	3 (2%)	4 (2%)	32 (5%)	-
Other	7 (4%)	8 (3%)	86 (12%)	-
Small Cell Carcinoma	19 (10%)	24 (10%)	66 (9%)	-
Squamous Cell Carcinoma	57 (31%)	72 (29%)	152 (22%)	-
Unknown or Not reported	4 (2%)	15 (6%)	3 (<1%)	-
Missing	0 (0%)	0 (0%)	5 (<1%)	-

Footnotes:
* National Lung Cancer Screening Trial (NLST) data subset to eligible NLST participants in LDCT Arm only.
† Report percent (%) among those with a reported value;

Supplementary Table S3. Test result by Lung-RADS score among non-cancer controls

Supplementary Table S3. Test result by Lung-RADS score among non-cancer controls			
**Enrollment CT Scan Result**	**Test Positive (n=63)**	**Test Negative (n=71)**	**Specificity (95% CI)**
Lung-RADS 1 (no nodules or nodules with specific calcifications)	19 (30%)	18 (25%)	0.49 (0.33, 0.64)
Lung-RADS 2 or nodule(s) <6 mm if no Lung-RADS	37 (59%)	49 (69%)	0.57 (0.46, 0.67)
Lung-RADS 3-4 or nodule(s) ≥6 mm if no Lung-RADS	3 (5%)	3 (4%)	0.50 (0.19, 0.81)
Lung-RADS and nodule size unknown or not reported	4 (6%)	1 (1%)	0.20 (0.04, 0.62)

Supplementary Table S4. Additional Population Health Simulation Scenarios

**Supplementary Table S4. Additional Population Health Simulation Scenarios**											
	**Predicted Screen Detected Cancer**		**Predicted Stage I Cancer Detected**		**Predicted Stage IV Cancer Detected**		**Predicted Deaths Averted****		**Predicted Number Needed to Screen*****		
Scenario	n	% Δ	%	Abs Δ	%	Abs Δ	n	Abs Δ	n	Δ	% Δ
1. LDCT Alone	24,489	-	26.20%	-	45.00%	-	128,287	-	202	-	-
2. Blood Test Low Uptake (100, 0)*	70,543	188.06%	31.8	5.6	39.8	-5.2	123,534	-9,473	140	-62	-30.69%
3. Blood Test High Uptake (100, 0)*	114,158	366.16%	37.9	11.7	34.7	-10.3	115,795	-17,212	127	-75	-37.13%
4. Blood Test Low Uptake (70, 20)*	60,035	145.15%	30.3	4.1	41.4	-3.6	124,901	-8,106	162	-40	-19.80%
5. Blood Test High Uptake (70, 20)*	93,205	280.60%	34.5	8.3	37.3	-7.7	120,985	-12,022	154	-48	-23.76%
6. Blood Test Low Uptake (50/50)*	53,895	120.08%	30	3.8	42	-3	123,885	-9,122	201	-1	-0.50%
7. Blood Test High Uptake (50/50)*	80,430	228.43%	33.2	7	38.7	-6.3	121,245	-11,762	204	2	0.99%
8. Blood Test Low Uptake (50, 10)*	49,395	101.70%	29.3	3.1	42.3	-2.7	126,330	-6,677	164	-38	-18.81%
9. Blood Test High Uptake (50, 10)*	71,835	193.34%	32.1	5.9	39.4	-5.6	122,460	-10,547	154	-48	-23.76%
10. Blood Test Low Uptake (25, 10)*	38,130	55.70%	28	1.8	43.5	-1.5	127,215	-5,792	180	-22	-10.89%
11. Blood Test High Uptake (25, 10)*	48,945	99.87%	29.2	3	42.2	-2.8	124,185	-8,822	177	-25	-12.38%

12. Blood Test Low Uptake (25, 0)*	35,895	46.58%	27.9	1.7	43.9	-1.1	126,600	-6,407	172	-30	-14.85%
13. Blood Test High Uptake (25, 0)*	45,795	87.00%	29.1	2.9	42.3	-2.7	123,630	-9,377	160	-42	-20.79%
14. Blood Test Low Uptake (10, 0)*	28,620	16.87%	27.3	1.1	44.5	-0.5	127,770	-5,237	190	-12	-5.94%
15.Blood Test High Uptake (10, 0)*	32,430	32.43%	27.8	1.6	43.5	-1.5	127,350	-5,657	182	-20	-9.90%
16. Blood Test Low Uptake (5, 0)*	27,135	10.80%	26.9	0.7	44.8	-0.2	128,205	-4,802	192	-10	-4.95%
17. Blood Test High Uptake (5, 0)*	32,250	31.69%	27.8	1.6	43.7	-1.3	127,725	-5,282	183	-19	-9.41%

Footnotes:
∆ Change from “base case” of LDCT Alone
% ∆ Percent change from “base case” of LDCT Alone
Abs ∆ Absolute change form “base case” of LDCT Alone
* Percent (%) of those individuals with positive test going on to get LDCT/ Percent (%) of those individuals with a negative test going on to get LDCT (e.g., 100, 0 is 100% of individuals with positive test go on to get LDCT and 0% of individuals with negative test go on to get LDCT)
Abs change in deaths from no-screening (n=133,007) * NNS, Number Needed to Scan with LDCT to Detect 1 Lung Cancer
